# A Dictyostelium model for BPAN disease reveals a functional relationship between the WDR45/WIPI4 homolog Wdr45l and Vmp1 in the regulation of autophagy-associated PtdIns3P and ER stress

**DOI:** 10.1080/15548627.2021.1953262

**Published:** 2021-07-27

**Authors:** Alba Tornero-Écija, Luis-Carlos Tábara, Miranda Bueno-Arribas, Laura Antón-Esteban, Cristina Navarro-Gómez, Irene Sánchez, Olivier Vincent, Ricardo Escalante

**Affiliations:** C.S.I.C./U.A.M., Instituto De Investigaciones Biomédicas Alberto Sols, Madrid, Spain

**Keywords:** Atg18, autophagosome, membrane contact site, omegasome, proppin proteins

## Abstract

PROPPINs are conserved PtdIns3P-binding proteins required for autophagosome biogenesis that fold into a characteristic group of seven-bladed beta-propellers. Mutations in WDR45/WIPI4, a human member of this family, lead to BPAN, a rare form of neurodegeneration. We have generated mutants for the two PROPPIN proteins present in the model system *Dictyostelium discoideum* (Atg18 and Wdr45l) and characterized their function. Lack of Wdr45l greatly impairs autophagy, while Atg18 only causes subtle defects in the maturation of autolysosomes. The strong phenotype of the Wdr45l mutant is strikingly similar to that observed in *Dictyostelium* cells lacking Vmp1, an ER protein required for omegasome formation. Common phenotypes include impaired growth in axenic medium, lack of aggregation, and local enrichment of PtdIns3P as determined by the use of lipid reporters. In addition, Vmp1 and Wdr45l mutants show a chronically active response to ER stress. For both mutants, this altered PtdIns3P localization can be prevented by the additional mutation of the upstream regulator Atg1, which also leads to recovery of axenic growth and reduction of ER stress. We propose that, in addition to an autophagy defect, local autophagy-associated PtdIns3P accumulation might contribute to the pathogenesis of BPAN by disrupting ER homeostasis. The introduction of BPAN-associated mutations in *Dictyostelium* Wdr45l reveals the impact of pathogenic residues on the function and localization of the protein.

## Introduction

Autophagy is an intracellular degradation pathway of proteins and organelles that serves as a mechanism for cellular cleaning and recycling, in addition to providing necessary nutrients during starvation. Macroautophagy (referred simply as autophagy hereafter) is characterized by the formation of a double membrane vesicle, the autophagosome, that engulf a wide variety of cargos, from nonselective cytosolic material (bulk autophagy) to specific organelles or protein aggregates (selective autophagy). Autophagy is a complex process that involves the hierarchical function of proteins that were initially identified in yeast, the so-called Atg proteins. These proteins regulate the different stages of autophagosome formation: the induction and generation of the initial membrane, known as the phagophore, the elongation and closure of the phagophore to form the autophagosome, and the fusion of the autophagosome with lysosomes [1,2]. In recent years, the autophagy-lysosome pathway has attracted considerable medical interest due to its involvement in various diseases, including neurodegenerative disorders, where autophagy dysfunction results in the accumulation of protein aggregates and abnormal mitochondria [3,4].

The most upstream signal for autophagy initiation is the activation of the Atg1/ULK complex by TORC1 and AMPK, master regulators of the nutritional and energetic status of the cells [5]. The Atg1/ULK complex is formed by Atg1, Atg13, Atg17, Atg29 and Atg31 in yeast, and by ULK1/2, ATG13, ATG101, and RB1CC1/FIP200 in mammalian cells. Once activated, the Atg1/ULK complex is recruited to the endoplasmic reticulum (ER) through interaction with VAPs (VAMP associated proteins) [6], and phosphorylates ATG9-containing vesicles, thought to be the seed membrane that forms the phagophore [7–10]. The Atg1/ULK complex also phosphorylates components of a specific phosphatidylinositol 3-phosphate (PtdIns3P) kinase complex (composed of Vps34, Vps15, Vps30/Atg6, Vps38 and Atg14 in yeast and PIK3C3/VPS34, PIK3R4/VPS15, BECN1/Beclin1, NRBF2 and ATG14 in mammals) [11]. The phosphatidylinositol 3-kinase Vps34 generates the phospholipid PtdIns3P by phosphorylation of phosphatidylinositol (PtdIns) in the nascent phagophore membrane and the ER-derived specialized domain known as the omegasome [8,12]. PtdIns3P recruits a family of proteins called PROPPINs (beta-propellers that bind polyphosphoinositides) that are essential for the further recruitment of other autophagic proteins. These proteins have a characteristic structure of WD-40 repeats that forms a domain known as seven bladed beta-propeller and contain two PtdIns3P-binding sites surrounding the conserved L/FRRG motif [13–17]. PtdIns3P also plays key roles in endocytic trafficking, where it recruits proteins containing the FYVE (Fab1, YOTB, Vac1 and EEA1) motif that specifically recognize PtdIns3P [18].

*Saccharomyces cerevisiae* has three PROPPIN proteins: Atg18, Atg21 and Hsv2. Atg18 and Atg21 play a key role in autophagy by mediating the recruitment of other Atg proteins such as Atg2 [19] and the conjugation machinery that will link Atg8 (LC3 in mammals) to the phagophore membrane [20–22], an essential step for the elongation and closure of the phagophore. There are four mammalian PROPPIN proteins known as WIPI1 (WD repeat protein domain, phosphoinositide interacting 1), WIPI2, WDR45/WIPI4 (WD repeat domain 45) and WDR45B/WIPI3. They appear to have a complex non-redundant hierarchical relationship that is not clearly established [23]. Mutations in one of the four human PROPPINs (WDR45) cause beta propeller protein-associated neurodegeneration (BPAN) [24,25]. This disease causes static encephalopathy in childhood and neurodegeneration in adulthood (SENDA), a form of neurodegeneration with iron accumulation in the brain (NBIA) [26,27]. The molecular and cellular mechanism of the disease is not fully understood. Defective autophagy was observed in lymphoblastoid cell lines derived from BPAN patients [25]. In addition, defective autophagic flux was reported in neurons of brain-specific knockout (KO) mice, a model that recapitulates some of the human symptoms, such as cognitive impairment and aberrant axonal function [28]. Although autophagy is likely to be involved due to the prominent role of WDR45 in this pathway, defects in ER homeostasis have also been observed in a KO mouse model of the disease [29].

Yeast Atg2, and *C. elegans* and mammalian homologs, ATG-2 and ATG2A/B, interact with the PROPPIN proteins, and this interaction is necessary to stabilize the localization of ATG2 in the PtdIns3P-enriched membranes at the phagophore assembly site [17,30–33]. Recently, several reports have shown that yeast Atg2 and mammalian ATG2A/B proteins have lipid transfer activity [34–37]. The current model proposes that the phagophore membrane acquires most of its lipids from ER due to the lipid transfer activity of the ATG2 proteins [2].

VMP1 (vacuole membrane protein 1) is a multispanning ER-membrane protein with a general function at various ER-organelle membrane contact sites (MCS) [38,39]. VMP1 has been proposed to regulate local changes in calcium concentration at MCS by activating the sarco-endoplasmic reticulum calcium ATPase [40]. In the absence of VMP1, local calcium levels rise and MCS become abnormally large but inactive. VMP1 must contribute in some way to the correct assembly of a functional MCS that allows the transfer of lipids between the ER membrane and those of other organelles. Particularly in autophagy, VMP1 regulates the level of PtdIns3P and the accumulation of lipid biosynthetic enzymes at the site of autophagosome formation. In the absence of VMP1, PtdIns3P levels increase locally and the omegasome becomes larger in mammalian cells, *C. elegans* and *Dictyostelium* [39–44]. Interestingly, all components of the autophagic machinery accumulate at the site of autophagosome formation, but the elongation of phagophores is impaired [39,42,45]. Thus, VMP1 is essential for the correct formation of a functional ER-phagophore MCS, probably by allowing the correct localization or function of VAPs and lipid synthesizing enzymes, although the precise mechanism is still unknown [38].

The autophagic machinery is very well conserved in all eukaryotes including *Dictyostelium discoideum*, a social amoeba that is gaining importance in the study of the basic mechanisms of human diseases. The autophagy pathway in *Dictyostelium* and mammalian cells is remarkably similar [46–59]. Here we investigate the consequence of the deletion of the two PROPPIN genes in *Dictyostelium* and study in more detail Wdr45l, the protein most similar to human WDR45. The introduction in the *Dictyostelium* protein of several pathological mutations inactivates the protein, further validating the use of *Dictyostelium* as a model of the disease. The striking phenotypic similarity between the Wdr45l mutant and the previously characterized Vmp1 mutant suggests that both proteins regulate the same autophagosome formation pathway. Interestingly the lack of Vmp1 and Wdr45l leads to chronic ER stress due to the abnormal localization of PtdIns3P. We propose that autophagy and ER-stress may contribute to the pathological mechanism of BPAN.

## Results

### The PROPPIN proteins in Dictyostelium discoideum

**Figure 1.**
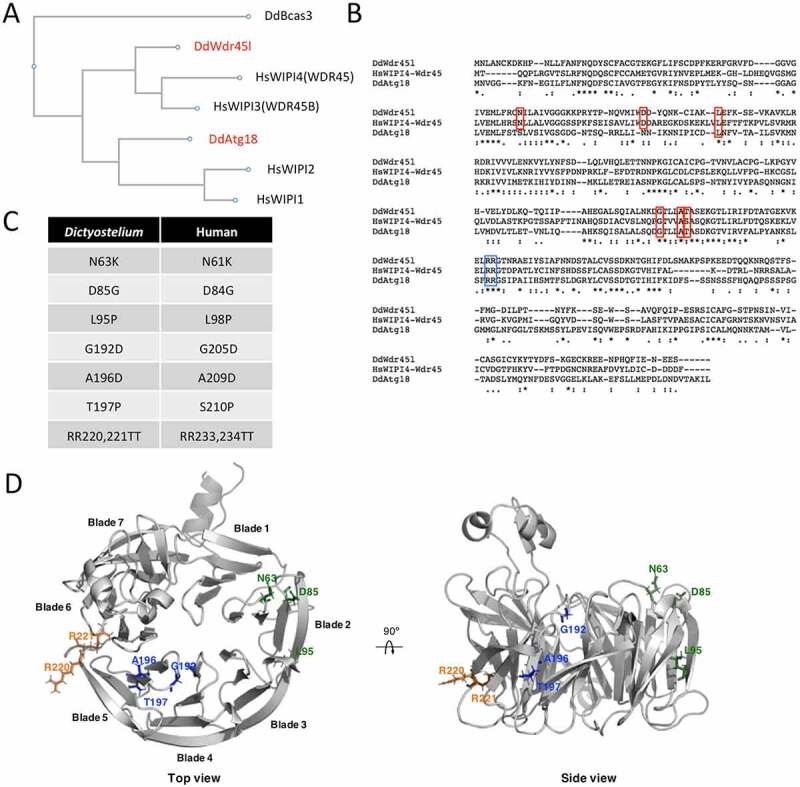
*Dictyostelium* has two PROPPIN proteins.

The genome of the social amoeba *Dictyostelium discoideum* encodes two PROPPIN proteins with strong similarity to the human WIPI family of proteins, which have been annotated as Atg18 and Wdr45l in DictyBase [60]. A CLUSTAL alignment of the *Dictyostelium* and human protein sequences was performed, and the resulting phylogenetic tree is shown in Figure 1a. The distantly related *Dictyostelium* PROPPIN Bcas3 was included in the phylogenetic analysis as an outgroup [61]. As previously proposed, the human proteins form two groups: WIPI1-WIPI2 and WDR45B-WDR45 [62]. While *Dictyostelium* Atg18 is phylogenetically related to the first group, Wdr45l belongs to the second group. An alignment of the two *Dictyostelium* PROPPINs and human WDR45 sequences confirms the higher conservation between Wdr45l and WDR45 that includes several amino acids mutated in BPAN patients [24] (marked in red in Figure 1b). These analyses suggest that *Dictyostelium* Wdr45l is more closely related to the human WDR45B and WDR45 proteins. The pathological mutations studied in this work and their positions in the amino acid sequence of WDR45 and Wdr45l are shown (Figure 1c). Note that the serine (S) amino acid at position 210 of the human protein corresponds to threonine (T) in the *Dictyostelium* protein. Since both residues have similar biochemical characteristics, the mutation affecting this position was also included in the studies described below. We generated a structure prediction of *Dictyostelium* Wdr45l to study the localization of the pathological mutations (Figure 1d). The structure of Atg18 from *Pichia angusta* was used as a template to model the *Dictyostelium* protein using the Robetta on-line service [63]. This prediction confirms that *Dictyostelium* Wdr45l shows the characteristic seven-bladed beta-propeller fold. The first three mutations are located in blade 2, whereas the other three localize in blade 5. The conserved L/FRRG motif is located between blades 5 and 6. It has been established that each R residue associates with a pocket (one in blade 5 and another in blade 6) to form two phosphoinositide binding sites [13,14,17].

The amino acid sequences of *Dictyostelium* Atg18 and Wdr45l were compared to the human proteins WIPI1, WIPI2, WDR45B and WDR45 and the *Dictyostelium* protein Bcas3. Phylogenetic analysis (**a**) and ClustalW alignment (**b**) were performed at https://www.genome.jp/tools-bin/clustalw. Red boxes mark the mutated amino acids in BPAN patients. Blue box marks the conserved RR motif involved in phosphatidylinositol binding. (**c**) Missense mutations in WDR45 identified in BPAN patients are shown together with the equivalent positions in the *Dictyostelium* protein Wdr45l. (**d**) Wdr45l tertiary structure was predicted with Robetta (http://robetta.bakerlab.org) using the crystal structure of Atg18 from *Pichia angusta* (PDB: 5LTD). Top and side views of Wdr45l were generated with PyMol software (PyMOL Molecular Graphics System, Version 2.4.0 Schrödinger, LLC). The amino acids associated with BPAN are shown in green and blue, and the conserved RR motif in yellow.

**Figure 2.**
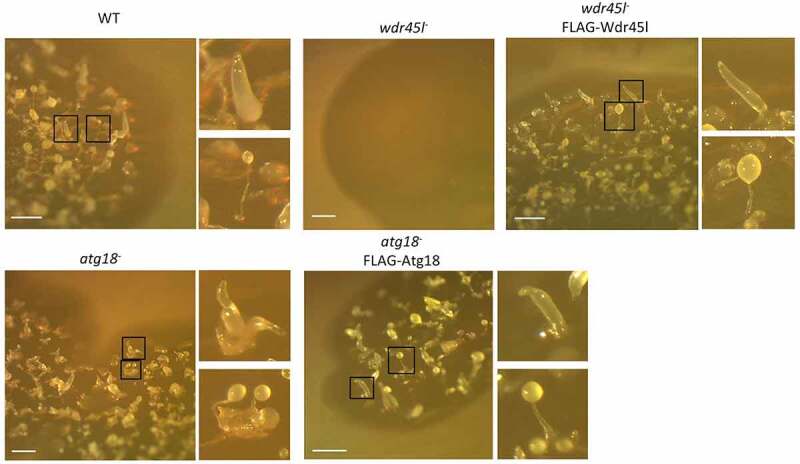
Developmental phenotype of *Dictyostelium* Atg18 and Wdr45l mutants.

It has been proposed that the different human PROPPINs have distinct but also redundant functions [64]. The presence of a single *Dictyostelium* protein in each paralog group may facilitate the study of this family of proteins and their functions. To this end, the genes encoding Atg18 and Wdr45l were disrupted by homologous recombination, which resulted in the insertion of a blasticidin-resistance cassette into the coding sequence of each gene. Both disruption cassettes were assembled via yeast gap-repair cloning, a technique that greatly facilitates the generation of KO *Dictyostelium* strains. Fig. S1 shows a schematic diagram of the technique, and Fig. S2 the specific constructs and the location of the insertions as well as the oligonucleotides used to identify the clones carrying the correct insertion. Many independent clones with similar phenotypes were isolated and one clone was selected for each gene for further studies. While the Wdr45l mutant showed a strong developmental phenotype with a lack of aggregation in SM plates in association with bacteria, the mutant Atg18 formed fairly normal-looking structures, although some of them appeared grouped and irregularly shaped compared with the WT. The phenotypes of both mutants were suppressed by the expression of their respective proteins fused to the FLAG epitope (Figure 2). Similar results were obtained in synchronous development on KK2 plates, although the phenotype of the Atg18 mutant was milder under these conditions (Fig. S3).

The indicated strains grew and developed in SM plates in association with *Klebsiella aerogenes* for 6 d. General views of the plaques and representative details of fingers and fruiting bodies are shown. WT develops into well-proportioned fingers and fruiting bodies. The Wdr45l mutant is unable to aggregate and Atg18 mutant shows clustered and misshapen structures. Both phenotypes are recovered upon expression of their respective proteins fused to the FLAG epitope. Bar: 1 mm.

### Autophagy is blocked in the Wdr45l mutant and only marginally affected in the Atg18 mutant

Next, we investigated autophagy in the newly generated mutants. For this purpose, we adapted for *Dictyostelium* a recently developed marker used to monitor autophagy in mammalian cells, GFP-Atg8-RFP-Atg8ΔG [65]. When expressed, this recombinant protein is cleaved by the Atg4 protease that releases equimolar amounts of GFP-Atg8 and RFP-Atg8ΔG. Only the first one can be conjugated to autophagic membranes, since the second one lacks the essential glycine (G) residue for covalent bonding to phosphatidylethanolamine. Therefore, the presence of green-only puncta unequivocally marks autophagic structures that are distinct from abnormal aggregates often formed in *Dictyostelium* autophagic mutants, as these aggregates contain both green and red markers (Fig. S4A). To validate this new marker we used WT and mutant cells lacking Atg1 or Vmp1, two well-characterized mutants deficient in autophagy [55,59] (Fig. S4B-D). Cells were maintained under growth conditions (HL5), or under starvation conditions (PDF) to induce autophagy. Both mutant strains have a blockade of autophagy that leads to the accumulation of large aggregates stained green+red in HL5 (similar results were obtained in PDF, data not shown), and lack the normal pattern of green-only tiny puncta observed in WT cells incubated in PDF. An added value of this marker is that it allows a more accurate assessment of the step in which autophagy is impaired. It is well known that the lack of Atg1 blocks autophagy at an early stage, preventing the lipidation of Atg8 to the phagophore membranes [42], and consistently, no trace of green-only staining was detected with this marker in this mutant. However, as Vmp1 functions downstream of Atg1 and Atg8, cells lacking Vmp1 accumulate abnormal and incomplete phagophores containing lipidated Atg8 in both *Dictyostelium* and mammalian cells [41,43,55]. Therefore, the use of this marker in Vmp1-deficient cells led to green+red aggregates often surrounded by large green-only structures that likely labeled abnormal phagophores (Fig. S4D).

**Figure 3.**
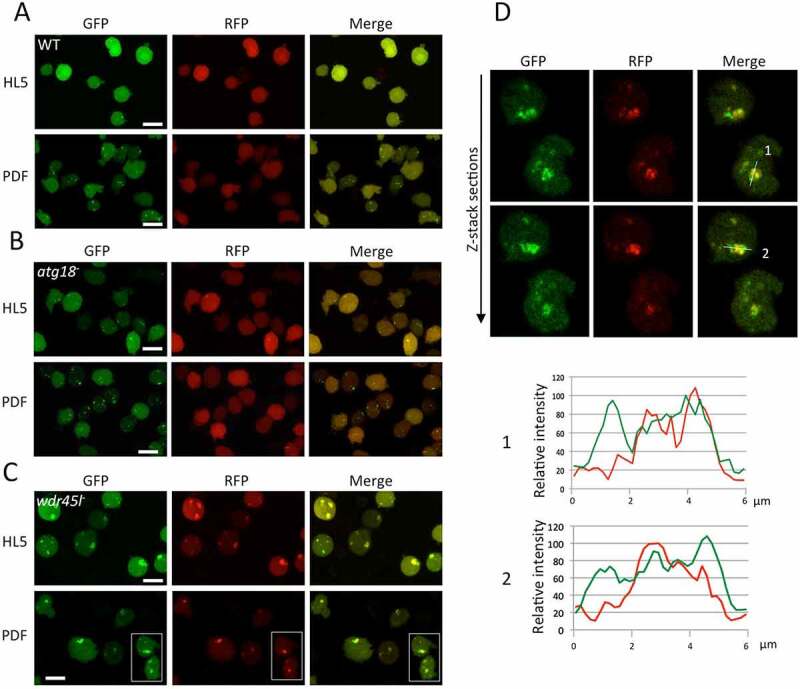
Analysis of autophagy by confocal microscopy.

Atg18 and Wdr45l mutants were transfected with this marker and visualized by confocal microscopy *in vivo* under growth (HL5) and starvation (PDF) conditions (Figure 3). WT cells showed tiny green-only puncta that increase in number with starvation, and a similar pattern was observed in cells lacking Atg18, suggesting a normal autophagosome formation in this mutant (Figure 3(a,b)). Few green+red dots could be seen sporadically, probably marking protein aggregates. This result is consistent with the mild developmental phenotype of the strain lacking Atg18. Conversely, Wdr45l-deficient cells lacked green-only puncta but accumulated large green+red aggregates under growth and starvation conditions, an indication of a blockade of autophagy (Figure 3c). Interestingly, as described above for the Vmp1 mutant, large green-only staining was also associated with the aggregates (Figure 3d), suggesting that Wdr45l may function, as Vmp1, in a step that once blocked leads to the accumulation of abnormal autophagic structures containing lipidated Atg8.

The WT strain and the indicated mutant strains were transformed with the autophagosome marker GFP-Atg8-RFP-Atg8-Δ-G and were visualized *in vivo* under growth (HL5) or starvation (PDF) conditions. (**a** and **b**) WT and the Atg18 mutant display a normal pattern of green-only puncta that is induced under starvation conditions. Only maximal projections are shown, since the colocalization analysis is not necessary due to the lack of red puncta. In the case of the Wdr45l mutant, maximum projections and z-stack sections are shown (**c**). Cells accumulate large green+red aggregates surrounded by abnormal green-only staining. (**d**) Enlarged sections of the cells marked in panel C are shown. Plot profiles were delineated following the white line depicted in the merged pictures. Bar: 10 μm.

**Figure 4.**
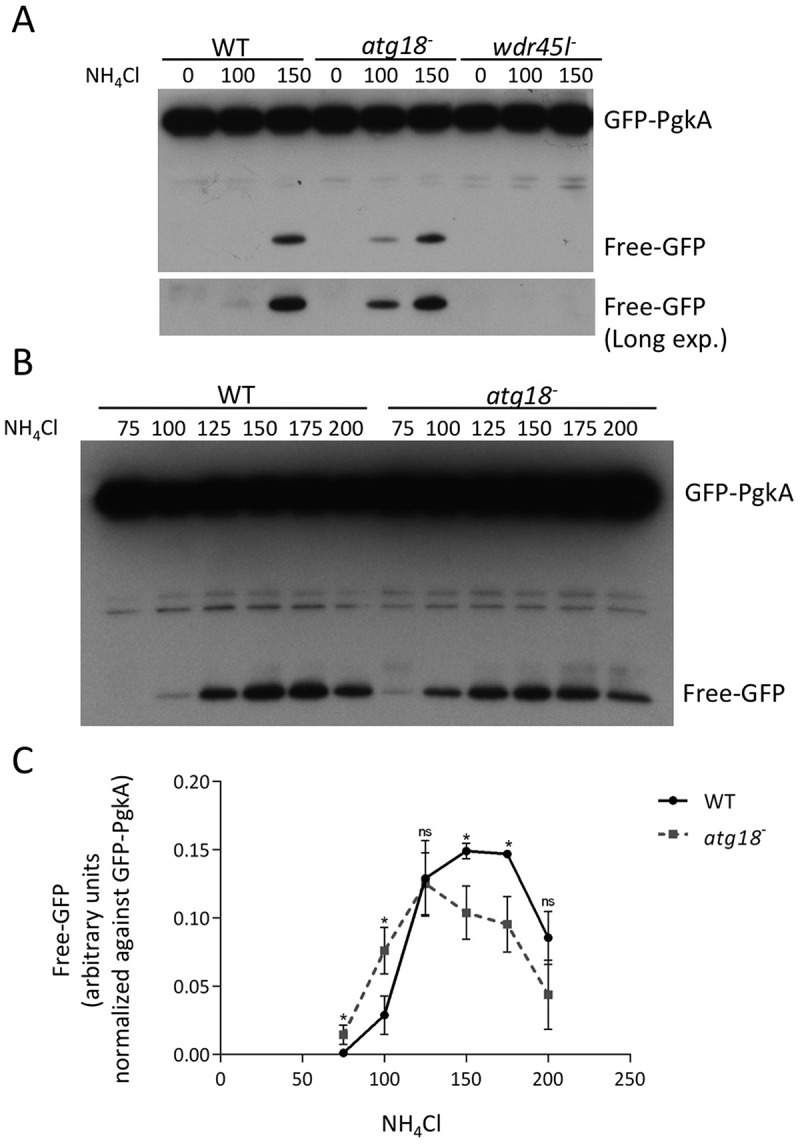
Quantitative analysis of autophagy using a proteolytic cleavage assay.

In order to characterize autophagy quantitatively in these strains, a complementary assay was performed based on the autophagic proteolytic degradation of GFP-PgkA, a bulk autophagy cargo. This technique was previously described and validated, allowing a quantitative assessment of autophagic flux [66,67]. NH_4_Cl is used in this assay to increase lysosomal pH and slow down degradation. A 100–200 mM window is needed to detect degradation that is otherwise too robust to allow free-GFP accumulation [66]. As shown in Figure 4a, similar levels of free-GFP were detected in the WT strain and the Atg18 mutant, while free GFP was undetectable in the Wdr45l mutant even after long time exposure of the western blot. This assay confirmed the minor effect of Atg18 deletion on bulk autophagy, while the absence of Wdr45l led to a blockade of autophagy. A thorough examination of the western blots showed a reproducible increase in free-GFP levels at 100 mM NH_4_Cl in cells lacking Atg18 (Figure 4a). Since a change in the range of NH_4_Cl required to detect the proteolysis of the marker may reflect alterations in lysosomal function, we further analyzed this possible difference using larger series of NH_4_Cl concentrations (Figure 4b). Under these conditions, free-GFP levels followed a curve that revealed the optimum concentrations of NH_4_Cl needed for maximum accumulation of free-GFP (Figure 4c). The decrease observed at large concentrations was due to the inhibition of proteolytic activity. We found a slight shift in the degradation curve in the strain lacking Atg18, since lower concentrations of NH_4_Cl were required to promote the accumulation of free-GFP and to achieve maximum response, suggesting that the degradative capacity of autolysosomes was slightly affected. This mild phenotype contrasts with the lack of aggregation phenotype and the blockade of autophagy in the strain lacking Wdr45l. These data, together with the sequence homology analyses described above, support the idea that Wdr45l is the ortholog of mammalian WDR45/WIPI4, which led us to study its function in more detail.

(**a** and **b**) WT and mutant strains expressing the GFP-PgkA marker were incubated at different concentrations of NH_4_Cl (mM), and the protein extracts were analyzed by western blot with an antibody to detect GFP. No free-GFP signal was detected even after a long exposure of the western blot in the Wdr45l mutant strain. (**c**) Quantification of the free-GFP signal at different concentrations of NH_4_Cl (mM). The mean and standard deviation of three independent experiments are shown. Significance was calculated using Student’s t-test in planned comparisons within each treatment. (* p < 0.05).

### Wdr45l and Vmp1 mutants show striking similarities, including local PtdIns3P enrichment

**Figure 5.**
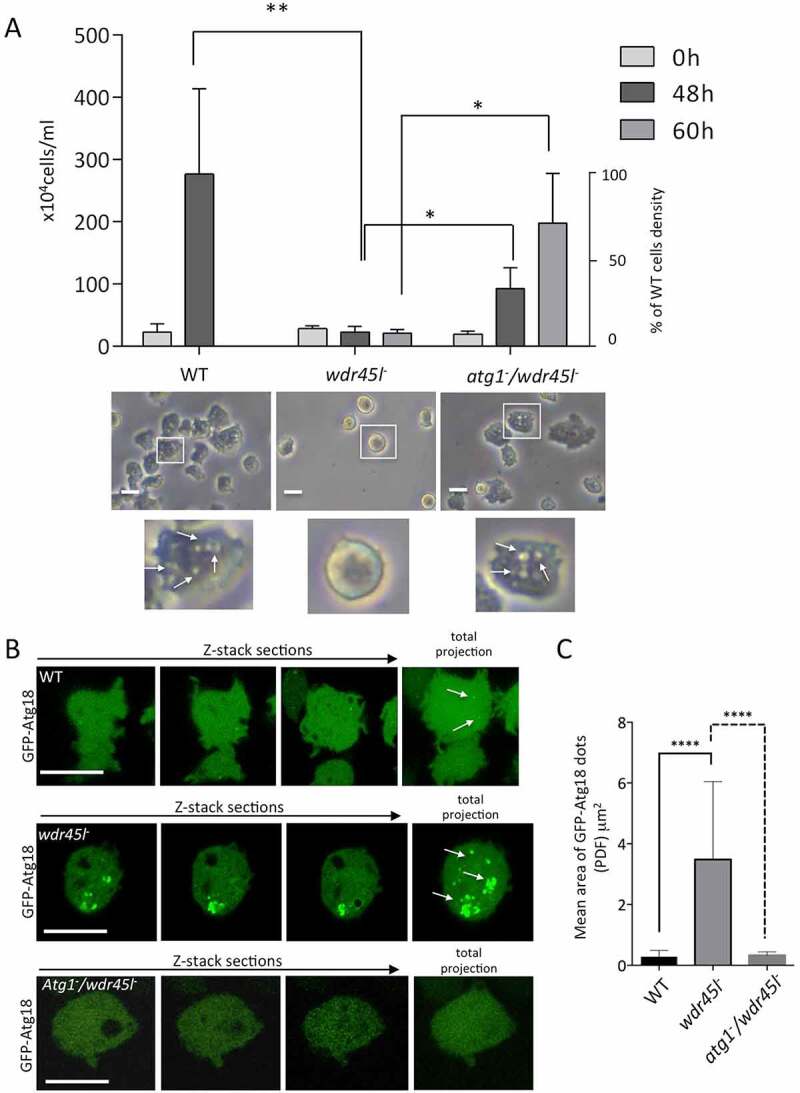
The Wdr45l mutant strain shows defects in axenic growth and PtdIns3P localization that are recovered in the Atg1/Wdr45l double mutant.

Comparison of the phenotype of the Wdr45l mutant with that of other *Dictyostelium* autophagic mutants led us to evaluate the similarities with the previously described Vmp1 mutant. *Dictyostelium* cells lacking Vmp1 are unable to aggregate, and display a blockade in autophagy [41,55,68], as also described for Atg1 and Atg13 mutants [54]. However, Vmp1 mutant has an additional defect in axenic growth that is not observed in Atg1, Atg13 or any other *Dictyostelium* autophagy mutant. This defect has been proposed to be associated with a local enrichment of the PtdIns3P signaling lipid at the site of autophagosome formation [41]. When PtdIns3P accumulation is abolished by the additional disruption of Atg1, the cells regain the ability to grow axenically. This epistatic relationship between Vmp1 and Atg1 suggests that abnormal autophagy-associated localization of PtdIns3P and/or downstream effectors has an effect on other cellular pathways that affects cell growth [41], although the underlying mechanism of growth inhibition is still unknown. Therefore, we compared all these phenotypes between the WT, the single Wdr45l mutant and the newly generated Atg1/Wdr45l double mutant. We found that growth in axenic media under shaking conditions was greatly impaired in Wdr45l-deficient cells (Figure 5a). Furthermore, under these conditions, mutant cells were more spherical and refringent under phase microscopy, making it difficult to discern internal vesicles. These macropinocytosis-derived vesicles are characteristically mobile and their presence was easily detectable by phase microscopy (Figure 5a, Movie S1 and S2). It is noteworthy that cells of the Wdr45l mutant can adhere to the plate in axenic media under submerged conditions, acquiring a more irregular shape (not shown). Interestingly, growth of the Atg1/Wdr45l mutant in axenic media was partially recovered, reaching 34% and 72% of the WT cell density at 48 and 60 h of growth, respectively. This was also accompanied by a recovery of cell morphology under phase contrast microscopy (Figure 5a).

(**a**) Cell growth and morphology in axenic media of the single and double mutants were analyzed after depositing the cells from SM plates in HL5 under shaking conditions. Both cell growth and morphology are partially recovered in the double mutant. Cell concentration was monitored at 0, 48 and 60 h and the mean with standard deviations is shown. Significance was calculated by Student’s t-test (* p < 0.05, ** p < 0.01). Representative pictures of cells from each strain are shown below and vesicles marked with arrows (see also Movie S1 and S2). Bar: 10 μm. (**b**) Cells expressing GFP-Atg18 were starved in PDF and observed under confocal microscopy. GFP-Atg18 localizes in autophagic structures enriched in PtdIns3P as previously described (see main text for details and also Fig. S5 for colocalization of this marker with the PtdIns3P reporter RFP-2xFYVE). WT cells show tiny puncta (marked with arrows), whereas mutant cells show enlarged signal (marked with arrows). See also Movie S3 and S4. This local enrichment is absent in the Atg1/Wdr45l double mutant. (**c**) The area occupied by the GFP-Atg18 marker in single and double mutants was measured in total projections of at least 50 cells in two independent experiments. Mean and standard deviation are shown. Significance of the difference was determined by one-way ANOVA followed by Dunnett´s multiple comparison tests (**** p < 0.0001). Bar: 10 μm.

We next examined whether the similarities between the Vmp1 and Wdr45l mutants included the abnormal localization of PdIns3P described previously for the Vmp1 mutant [41]. We used GFP-Atg18 as autophagic marker, which has been previously shown to mark PdIns3P-enriched autophagic structures [41,69]. We observed large and persistent accumulations of this marker in Wdr45l-deficient cells (Figure 5b, Figure 5c, Movie S3 and S4). To further confirm that GFP-Atg18 marks PtdIns3P-enriched regions, we used the reporter RFP-2xFYVE that labels PtdIns3P-positive endosomes and autophagosomes in *Dictyostelium* [41,70]. Colocalization of GFP-Atg18 with RFP-2xFYVE was observed in tiny puncta in WT and in the large accumulations marked by GFP-Atg18 in the Wdr45l mutant (Fig. S5). Note that RFP2xFYVE also labels endosomes that are not marked by the autophagy-specific marker GFP-Atg18. This abnormal accumulation of GFP-Atg18 was prevented in the Atg1/Wdr45l double mutant (Figure 5(b,c)). Altogether, these results strongly suggest that PtdIns3P is abnormally enriched at the autophagosome formation site, which could lead to altered recruitment of downstream autophagic proteins.

### Wdr45l and Vmp1 mutants show chronically activated ER-stress response that can be suppressed by Atg1 inactivation

**Figure 6.**
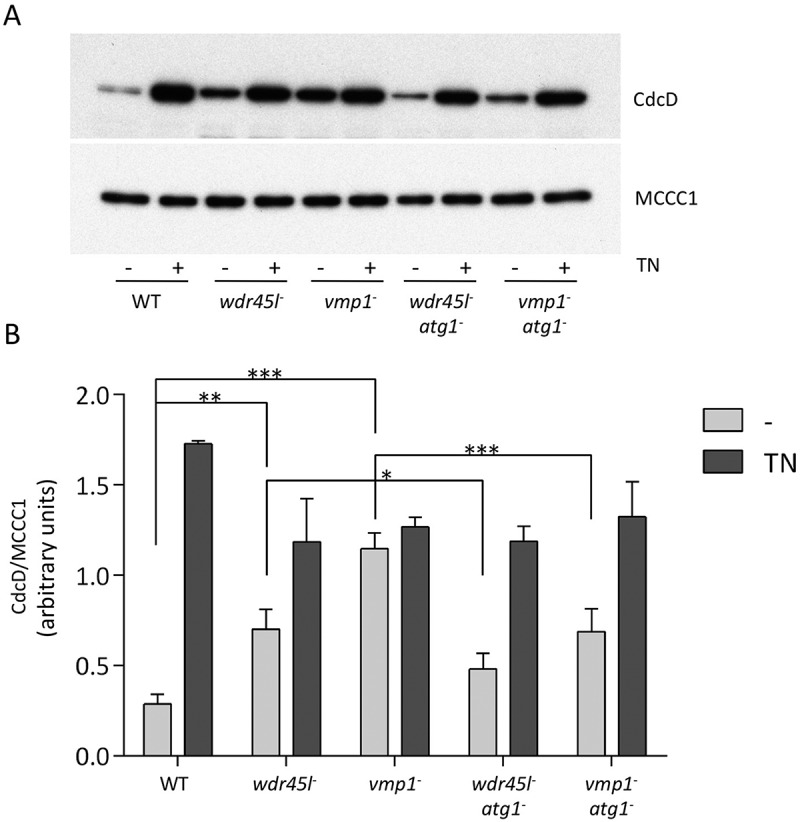
ER-stress response in cells lacking Wdr45l and Vmp1.

We next asked whether Vmp1 and Wdr45l mutants, because of their similar defects in autophagy and PtdIns3P localization, might have impaired ER homeostasis. The accumulation of unfolded proteins activates a complex ER stress pathway, the unfolded protein response (UPR), a process that has often been associated with neurodegenerative diseases [71]. In *Dictyostelium*, the UPR restores protein homeostasis by activating a gene expression program that inhibits general protein synthesis and cell growth, and increases the folding and degradation capacity of cells by activating the expression of chaperones and degradative enzymes [72,73]. ER-stress activation in *Dictyostelium* can be monitored by the increased levels of CdcD (similar to human transitional endoplasmic reticulum ATPase [VCP/p97] and yeast Cdc48) [72]. WT and the different mutant strains (Wdr45 and Vmp1 single mutants, and the double mutants with Atg1) were exposed to tunicamycin (TN) to induce ER-stress and CdcD levels were analyzed by western blot (Figure 6a). The levels of this marker increased after TN treatment (comparison between the light gray and gray bars in Figure 6b) in WT as previously described [72]. Notably, CdcD levels in the absence of TN (light gray bars) were significantly higher in Vmp1 and Wdr45l mutants compared to WT levels, and this effect was partially suppressed by Atg1 inactivation in both mutants. These results strongly suggest that *Dictyostelium* cells lacking Vmp1 or Wdr45l suffer a chronic ER-stress response associated with the abnormal PtdIns3P localization produced by the autophagy blockade in these mutants.

Cells from the different strains were incubated with or without tunicamycin (TN) and protein extracts were analyzed by western blot to quantify the levels of the ER-stress marker CdcD. MCCC1 was used as a loading control. TN treatment was used as a positive control of ER-stress response. A representative blot (**a**) and the quantification of three independent experiments are shown (**b**). Planned comparisons were performed to determine whether the single mutants have significantly higher levels of the ER stress marker with respect to WT (light gray bars), and whether the levels of the ER stress marker are reduced in the double mutants with respect to the single mutants. The mean and standard deviation are shown. The significance of such comparisons was determined by 2way ANOVA followed by Fisher Least Significance Difference Test (* p < 0.05; ** p < 0.01; ***p < 0.001).

### BPAN mutations disrupt Wdr45l localization and function

Most BPAN patients have truncating mutations or deletions in WDR45 that likely result in a loss of protein function, a situation that is equivalent to our KO mutant. In addition, as described earlier, there are several missense mutations in WDR45 that are also associated with BPAN, and most of the amino acids involved are conserved in *Dictyostelium* Wdr45l (Figure 1b). In order to study the consequences of such mutations on the function of the protein, the same mutations were introduced by site-directed mutagenesis in the *Dictyostelium* Wdr45l protein fused to the FLAG epitope to allow its detection by immunofluorescence. Figure 1c lists the pathogenic mutations used in this study and the corresponding conserved residues in *Dictyostelium* Wdr45l and human WDR45. In addition, the mutation in the conserved L/FRRG motif, which is essential for PtdIns3P binding, was included as a control [22,74,75].

**Figure 7.**
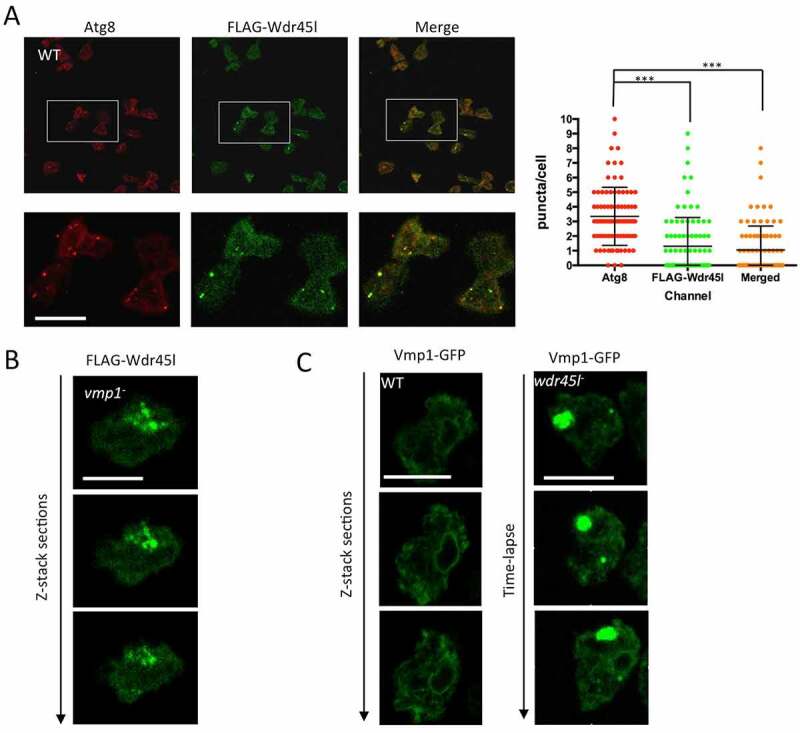
Subcellular localization of Wdr45l.

We first studied the localization pattern of WT FLAG-Wdr45l by immunofluorescence. This protein showed a punctate pattern that colocalized with the autophagic marker Atg8 in starvation conditions, confirming the presence of Wdr45l in autophagosomes (Figure 7a). The quantification of the level of colocalization showed that not all Atg8 puncta contain Wdr45l, suggesting that Wdr45l associate transiently with Atg8-positive autophagic structures. We studied whether this localization pattern could be affected by the lack of Vmp1. In Vmp1 mutant cells, FLAG-Wdr45l did not show the normal WT punctate pattern and accumulated in large structures (Figure 7b). Strikingly, the normal ER localization pattern of Vmp1-GFP was also reciprocally affected by the lack of Wdr45l, with the presence of large accumulations (Figure 7c and Movie S5), indicating a mutual dependence of both proteins for their correct subcellular localization.

(**a**) Wdr45l fused to the FLAG epitope were expressed in WT, starved for 2 h in PDF and fixed for immunofluorescence with FLAG and Atg8 antibodies. Colocalization between both proteins was analyzed by confocal microscopy. The number of puncta per cell was quantified from 89 cells from three independent experiments (at least 20 cells per experiment). The mean and standard deviation are shown, and the significance of comparisons was determined by Kruskal-Wallis test followed by Dunn´s multiple comparisons test (***p < 0.001). Bar: 10 μM. (**b**) Vmp1 mutant cells transformed with FLAG-Wdr45l were analyzed by immunofluorescence with anti-FLAG antibody. Wdr45l is accumulated in aberrant structures. Bar: 10 μm. (**c**) Video-lapse confocal analysis of Wdr45l mutant cells expressing Vmp1-GFP. The marker is abnormally accumulated in the mutant background (see also Movie S5). The normal localization pattern of Vmp1-GFP is shown in WT cells. Z-stack sections are displayed to show the absence of abnormal accumulations. Bar: 10 μm.

**Figure 8.**
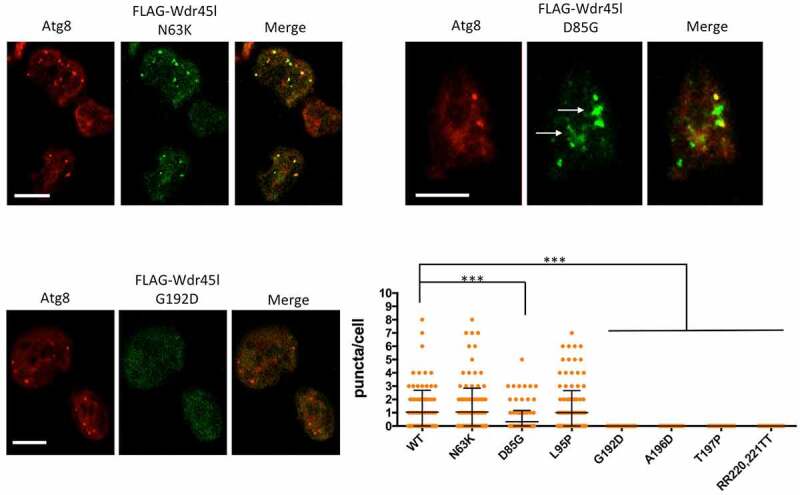
Subcellular localization of Wdr45l carrying pathological mutations.

Next, we analyzed the pattern of the mutated Wdr45l proteins. Figure 8 and S6 show representative cells analyzed by confocal microscopy and the graph shows a quantification of FLAG-Wdr45l puncta (green channel) that colocalize with the autophagosome marker Atg8 (red channel). Two of the mutants (N63K and L95P) were able to form puncta that colocalize with Atg8, as the WT protein, indicating that these mutations did not affect the localization of the protein. The mutant protein D85G, although capable of forming some puncta, had a reduced level of colocalization with Atg8. In addition, this mutant protein showed large accumulations, which is also indicative of an abnormal localization of the protein. The other mutants (G192D, A196D and T197P), as well as the negative control mutant designed to prevent PtdIns3P binding (RR231,232TT), did not show any punctate signal colocalizing with Atg8, indicating that these mutations impair the normal localization of the protein in autophagic structures. Western blot analysis of protein extracts shows that all the mutant proteins are expressed in the transformants (Fig. S7A). Although the levels of all mutant proteins are moderately reduced except for one (RR220-221TT), they are sufficient for immunolocalization studies. It is conceivable that some mutations affect protein folding or stability, causing changes in steady-state levels.

Mutant forms of Wdr45l fused to the FLAG epitope were expressed in WT, starved for 2 h and fixed for immunofluorescence with FLAG and Atg8 antibodies. Colocalization between both proteins was analyzed by confocal microscopy. For simplification only images of representative phenotypes are presented (N61K, D85G and G129D). The complete set of images can be seen in Fig. S6. White arrows in D85G mark aberrant accumulations of the protein. Three independent experiments were performed with similar results. In addition, DNA from independent clones of each transformation was isolated to verify “*a posteriori*” the presence of the expected mutations by sequencing PCR-derived *wdr45l* fragments. The number of puncta colocalizing with Atg8 was quantified and compared between all mutant proteins and the WT Wdr45l. At least 50 cells were used from each mutant. The mean and standard deviation are shown. The significance of comparisons was determined by Kruskal-Wallis test. (***p < 0.001). Bar: 10 μm.

**Figure 9.**
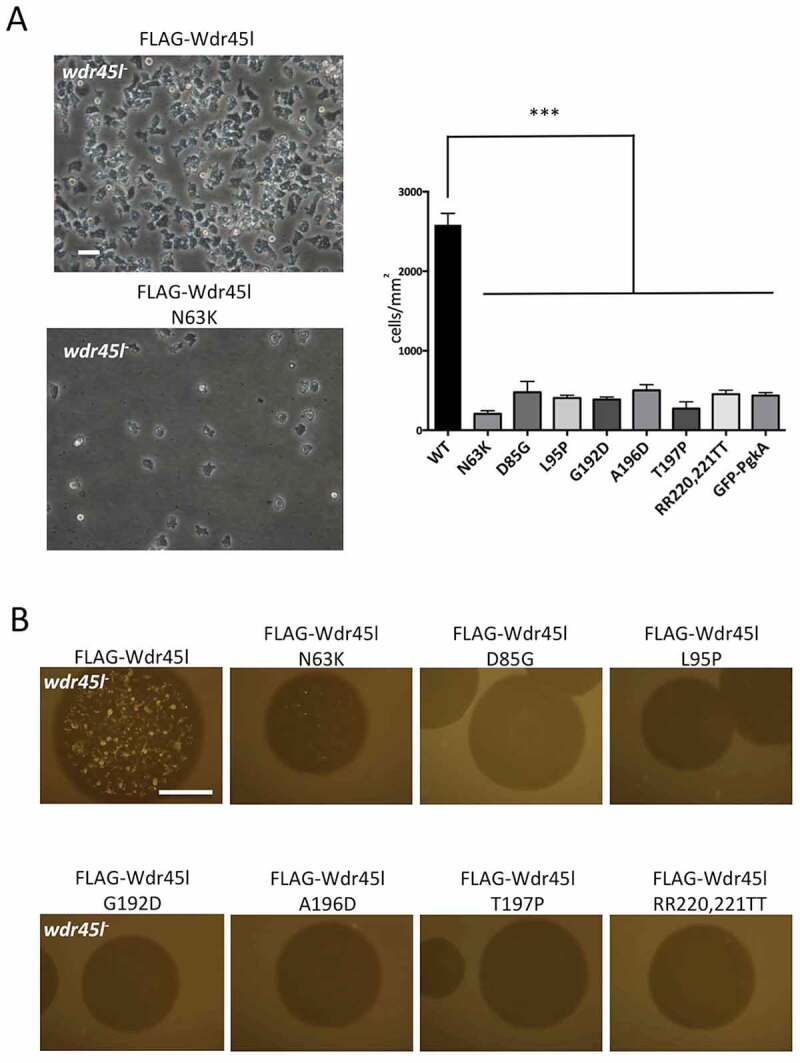
Functional analysis of the mutated forms of Wdr45l.

Besides protein localization, the severe phenotype of the Wdr45l mutant in the *Dictyostelium* model also allows a simple assessment of the impact of BPAN-associated mutations on the protein’s function. The WT and mutant derivatives of Wdr45l were transfected into the Wdr45l mutant strain. Transformants were selected in axenic culture and then plated in SM to assess the developmental phenotype. During selection in axenic culture, only the WT construct restored the normal growth and cell morphology (Figure 9a and S8). All the other transformants had similar poor growth and abnormal cell morphology, including a strain transformed with the marker GFP-PgkA, used as a control of the mutant phenotype. Similarly, development was only recovered in transformants expressing the WT form of Wdr45l (Figure 9b). A western blot analysis showed that all the mutant proteins were expressed in the transformants (Fig. S7B). Expression levels of most mutant proteins were also reduced in the Wdr45l mutant background. Perhaps the impaired autophagy and altered ER-stress response that occurs in this mutant affect the structure or stability of the mutant proteins even more than in the WT background. Therefore, we cannot rule out that the lack of functionality of the mutant proteins may be influenced by their reduced expression.

(**a**) WT and mutant forms of Wdr45l fused to the FLAG epitope were transformed in the Wdr45l mutant. After 7 d under selection, cells were observed by phase microscopy to assess growth and cell morphology. Two independent transformations were performed with similar results. In addition, DNA was isolated from independent clones of each transformation to verify “*a posteriori*” the presence of the expected mutations by sequencing PCR-derived *wdr45l* fragments. For simplification, only representative images of cells expressing the WT and the mutant N63K are shown. The complete set of images can be seen at Fig. S8. For quantification of cell growth, three random pictures of the plates were used to determine cell density (number of cells per mm^2^). The mean and standard deviation of WT and all the mutants are shown. Significance of the difference with the control WT sample was determined by one-way ANOVA followed by Dunnett´s multiple comparison tests. (***p < 0.001). Bar: 10 μm. (**b**) Transformants were plated in SM and development was assessed after 6 d. Two independent transformations were performed with similar results. In addition, DNA was isolated from independent clones of each transformation to verify “*a posteriori*” the presence of the mutations by sequencing PCR-derived *wdr45l* fragments. Bar: 10 mm.

In summary, our data suggest that the tested mutations, despite their different effect on subcellular localization, greatly affect the function or stability of the proteins and are therefore equivalent to the KO mutant. These results confirm the critical importance of these residues and further validate *Dictyostelium* as a model of the disease. Furthermore, our findings provide novel insights into the role of PROPPINs and reveal a functional relationship between Vmp1 and Wdr45l in a process that links autophagy dysfunction with ER stress that may play a role in BPAN disease.

## Discussion

### Dictyostelium has two PROPPIN proteins with distinct functions

The number of PROPPIN proteins varies in different species and they appear to have redundant and non-redundant functions in autophagy and endocytic trafficking. In autophagy, they play roles in different stages of the formation of autophagosomes. Due to this complexity, the functional equivalence of the different PROPPINs in different experimental models is not yet clear. We have shown here that Wdr45l is required for autophagy, while Atg18 appears to have only a marginal role in this process. Cells lacking Wdr45l have a blockade of autophagy flux as determined by the proteolytic cleavage assay but, importantly, lipidation of Atg8 can still be detected in abnormal structures surrounding protein aggregates. This strongly argues that Wdr45l operates at the same stage as the yeast PROPPIN Atg18 [19,22]. In mammalian cells, the proteins that best fit the function of yeast Atg18 and *Dictyostelium* Wdr45l are WDR45B and WDR45 [23]. In addition, phylogenetic analysis shows that *Dictyostelium* Wrd45l belongs to the group of WDR45-WDR45B and mutated residues in WDR45 in BPAN patients are conserved in the *Dictyostelium* protein.

The ability of cells to lipidate Atg8 in the absence of Wdr45l or Atg18 in *Dictyostelium* suggests that the lipidation machinery can be recruited to the phagophore in the absence of PROPPIN proteins in this organism. This is in apparent contrast with the specific function of one of the three yeast PROPPINs, Atg21, that mediates the recruitment of the Atg12–Atg5-Atg16 complex necessary for the subsequent lipidation of Atg8 to the phagophore membrane [20,22]. In mammalian cells, the same function is achieved by WIPI2 [76]. However, although the knockdown of WIPI1 or WIPI2 impairs the recruitment of ATG16L1 [77], LC3 lipidation is not completely prevented [78]. In addition, a recent report shows that the C-terminus of mammalian ATG16L1 mediates membrane binding and can sustain LC3 lipidation in the absence of WIPI2 [79]. Interestingly, while yeast Atg16 lacks this C-terminal domain present in mammalian ATG16L1, it is found in the *Dictyostelium* Atg16 homolog [49]. Thus, it is possible that *Dictyostelium* Atg18 is the functional counterpart of yeast Atg21 and mammalian WIPI2 but, as in mammals, the recruitment of the lipidation machinery is not strictly dependent on PROPPINs and therefore, the effect on bulk autophagy is only marginal in the absence of *Dictyostelium* Atg18. Nevertheless, we cannot rule out that PROPPIN-dependent recruitment of the lipidation machinery, although not essential in *Dictyostelium*, is necessary for optimal lipidation, which may explain the slight reduction of the autophagic flux observed in the mutant.

### New candidate genes for BPAN-like diseases?

In *Dictyostelium*, the range of developmental phenotypes associated with impaired autophagy varies in severity from abnormal fruiting body morphology, characterized by a multi-tipped phenotype and lack of sporulation, to the lack of aggregation [52,53,56,80]. The most severe phenotype occurs in cells lacking Atg1, Atg13, Vmp1 [41,55], and Wdr45l as described here. Although all these mutants have undetectable autophagy, we have shown here that Vmp1 and Wdr45l share additional and specific phenotypes, including growth impairment in axenic culture, accumulation of lipidated Atg8 and PtdIns3P, and an epistatic relationship with Atg1. This remarkable similarity strongly suggests that these two proteins work at the same stage of autophagosome formation, the elongation of the phagophores.

**Figure 10.**
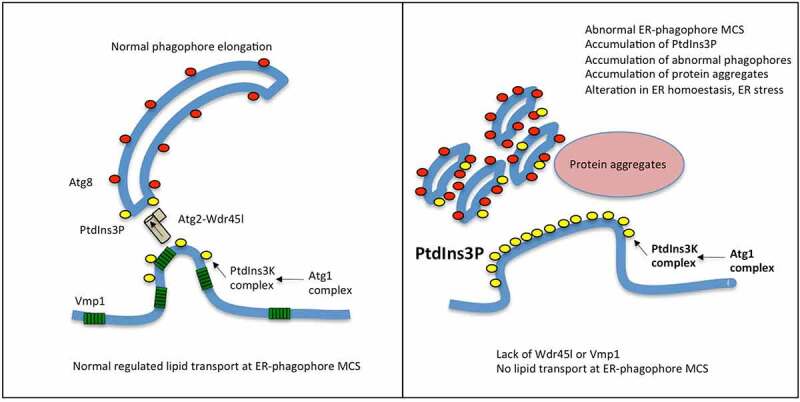
Working model of phagophore expansion and the function of Wdr45l and Vmp1.

Recent research has established a model that explains how the phagophore membrane is elongated. Lipids are transported from the ER to the phagophore by the lipid transfer protein ATG2, which forms a complex with the PROPPIN proteins Atg18 in yeast, and WDR45 in mammalian cells [2,10]. This occurs at the omegasome, a specialized ER-derived platform that establishes a close contiguity with the phagophore, providing the correct proximity and lipid environment to allow lipid transport [10,36,81,82], which is in essence, the definition of MCS. The correct morphology and function of the omegasome require the ER protein Vmp1, although the molecular function of this protein is still unknown [38–40,42]. This model would explain why the lack of Wdr45l and Vmp1 leads to similar phenotypes, since their absence would prevent lipid transport in a similar way (Figure 10). One consequence of this blockage is the persistent accumulation of signaling components such as PtdIns3P, the hypertrophy of omegasomes and the accumulation of abnormal lipidated phagophores. Interestingly a similar phenotype was also observed in the absence of ATG2 in HeLa cells, which led to the conclusion that both ATG2 and VMP1 works at the same stage [42]. Studies of mutants of the *C. elegans* WDR45 (EPG-6) and ATG2 homologs further support the role of both proteins in the progression from omegasomes to autophagosomes [31].

A working model of phagophore expansion based on the recent advances in the literature and the results from this work is shown. A functional MCS between the ER and the phagophore, the omegasome, is required for intermembrane lipid transport by ATG2 proteins. *Dictyostelium* and mammalian VMP1 proteins are required for the functionality of different ER-organelle MCS, including the ER-phagophore MCS, although the precise molecular function of this protein is unknown. Lipid transport could be compromised in cells lacking Vmp1 or Wdr45l, leading to similar phenotypes, including the abnormal localization of PtdIns3P, accumulation of abnormal autophagic structures and protein aggregates. This may lead to additional defects such as chronic ER stress and cell growth arrest in *Dictyostelium*. These defects can be prevented by inhibition of the upstream signaling protein Atg1. The link between aberrant autophagy-associated PtdIns3P signaling and the ER stress response, as well as the connection between Vmp1 and Wdr45l are novel findings that will warrant further study in the context of BPAN disease.

According to this model, BPAN-like diseases could arise from mutations in any component of the machinery involved in lipid transport during phagophore elongation, as described for the WDR45 mutations. Therefore, VMP1 and ATG2 may be candidate genes for involvement in idiopathic BPAN/NBIA-like diseases.

### An abnormal PtdIns3P localization is associated with ER stress

Our results suggest that local enrichment of autophagy-associated PtdIns3P and/or the altered recruitment of downstream autophagic proteins can lead to activation of the ER stress response in *Dictyostelium*, as observed in cells lacking Vmp1 or Wdr45l. Activation of an ER stress response in *Dictyostelium* may explain some of the additional phenotypes observed in the absence of these proteins. Previous studies have shown that the treatment of *Dictyostelium* cells with tunicamycin leads to cell growth arrest and changes in cell morphology with a reduction in pseudopodia and the acquisition of a spherical shape [72]. This ER stress response modulates the transcriptional levels of cytoskeleton and lipid metabolism genes, among others [72,73]. Growth arrest and spherical morphology could help cells to restore lipid and protein homeostasis by decreasing cellular activity and energy requirements.

Recently, ER-stress has been reported in a *wdr45* KO mouse [29]. It has been suggested that autophagy dysfunction in WDR45-deficient neurons leads to the accumulation of misfolded proteins that would eventually induce ER stress. Our results suggest that abnormal levels of autophagy-associated PtdIns3P, rather than lack of autophagy, triggers ER stress in the *Dictyostelium* model. This hypothesis is supported by the fact that double mutants Wdr45l/Atg1 and Vmp1/Atg1 decrease ER stress response and recover cell growth in the absence of autophagy. It would be interesting to determine if a similar mechanism exists in the cell types affected in BPAN disease. The local enrichment of PtdIns3P could be the result of increased levels and thus affects the downstream signaling dynamics of this lipid. If so, attenuation of PtdIns3P levels could be considered as a possible therapeutic target. The mechanism by which PtdIns3P activates the ER stress response will require further studies.

### Pathogenic missense mutations in *Dictyostelium* Wdr45l impair the function of the protein

The introduction of pathogenic mutations associated with BPAN in *Dictyostelium* Wdr45l shows that all these mutant proteins are unable to rescue the KO phenotype. Although the number of BPAN patients with missense mutations is very low, the available data also suggest that they are as severe as the truncated mutations with the exception of one patient with the D84G mutation who was very mildly affected [83]. Despite the phenotypic similarity, we have found that the pathogenic mutations N61K, D84G and L98P do not impair the localization of the protein in autophagic structures containing Atg8, although it is partially affected in D84G. These residues are located in blade 2 at the opposite site of the conserved FRRG motif. Blade 2 has been implicated in the interaction of Atg18 with Atg2 in yeast [32] and the interaction of mammalian WDR45 with ATG2A [30]. Therefore, although the protein localization is not altered, its function may be impaired by the inability to interact with Atg2. Following the same line of argument, the three pathological mutations G205D, A209D and S210P are located within the region that has been defined by structural analysis of yeast Hsv2 as one of the PtdIns3P binding sites along with one R residue of the conserved FRRG motif [13,14,17]. Our results support the idea that these residues probably affect PtdIns3P binding, since they are required *in vivo* for the correct localization of Wdr45l in Atg8-containing autophagic structures. In addition, the reduced expression levels of most mutant proteins in both the WT and mutant background suggest that these mutations could also affect protein folding or stability.

The results provided in this study highlight the interest of using the simple social amoeba *Dictyostelium* as a model for the study of autophagy-related diseases and BPAN in particular. The phenotypes associated with Wdr45l dysfunction, the relationship with Vmp1, and the link between PtdIns3P, autophagy, and ER stress warrant further investigation in the future. The optimization of various techniques for monitoring autophagy provided in this work will also reinforce the use of *Dictyostelium* in the field of autophagy.

## Materials and methods

### Strains, cell growth and transformation

The strains used in this work were derived from *Dictyostelium discoideum* strain AX4, named WT hereinafter (Dicty-stock center, 3,025,622). Cells were grown axenically in HL5 (Formedium, HLB0102) or in association with *Klebsiella aerogenes* in SM plates (10 g/l glucose [Merck, 15,639], 10 g/l peptone [Pronadisa, 1612.00], 1 g/l yeast extract [Conda, 1702.00], 0,5 g/l MgSO_4_, 1,9 g/l KH_2_PO_4_, 0,6 g/l K_2_HPO_4_ y 20 g/l agar [Intron Biotechnology. 25,999]) [84]. For axenic growth, cells were deposited in HL5 in suspension (shaking conditions) using an orbital shaker (135 rpm),

or without shaking in culture plates (submerged condition). For starvation, cells were resuspended in PDF buffer (20 mM KCl, 9 mM K_2_HPO_4_, 13 mM KH_2_PO_4_, 1 mM CaCl_2_, 1 mM MgSO_4_, pH 6.4). Transformations were carried out by electroporation as described previously [85]. Synchronous development was performed on KK2 agar plates as previously described [86].

The Vmp1 mutant strain was described previously [68]. Disruption of Atg1 in the WT strain was carried out by using a construct kindly provided by Dr. Jason King (Department of Biomedical Sciences, University of Sheffield, Sheffield, United Kingdom) [41]. For the generation of double mutants, the BSr cassette of the Atg1 mutant was first removed by the expression of the cre recombinase as described [87] and the strain was subsequently transformed with a disruption construct for Wdr45l (described below). For most procedures involving Vmp1 and Wdr45l mutants (due to their poor growth in axenic media), cells were grown in SM plates for 5–6 d, taken from the growing zone and resuspended in HL5 right before their use.

The yeast strain FY250 (MATa his3Δ200 leu21 trp1 Δ 63 ura3-52) was a gift from Dr. F. Winston (Harvard Medical School, Boston, MA, USA) and was used for the construction of disruption cassettes by gap-repair.

### Generation of disruption constructs by gap-repair

*Dictyostelium atg18* and *wdr45l* genes were disrupted by homologous recombination using a disruption construct generated by gap repair in *Saccharomyces cerevisiae*. For this, flanking regions of each gene were amplified by PCR from genomic DNA (see Fig. S1), using primers containing an additional sequence to allow homologous recombination with the plasmid and BS cassette. The general design of primers is as follows:

5ʹ-Flanking region (FR) 1, primer forward (1):

5ʹ-GGGAACAAAAGCTGGGTACCGGGCCCCCCC+specific sequence of the gene to be disrupted (up to 30 nucleotides)-3ʹ.

5ʹ-Flanking region (FR) 1, primer reversed (1ʹ):

5ʹ-CCCGGGAAGCTTATCGATACCGTCGACCTC+ specific sequence of the gene to be disrupted (up to 30 nucleotides)-3ʹ.

3ʹ-Flanking region (FR) 2, primer forward (2ʹ):

5ʹ-CATATGCCGCATGGTTAATTCCTGCAGCCC+ specific sequence of the gene to be disrupted (up to 30 nucleotides)-3ʹ.

3ʹ-Flanking region (FR) 2, primer reversed (2):

5ʹ-AATTGGAGCTCCACCGCGGTGGCGGCCGCT+specific sequence of the gene to be disrupted (up to 30 nucleotides)-3ʹ.

The blasticidine cassette containing loxP motifs were amplified from the pLPBLP plasmid (obtained from Dicty-stock center, ID-9) with the following primers:

OV911 (BS1): 5ʹ-GAGGTCGACGGTATCGATAAGC-3ʹ

OV912 (BS2): 5ʹ-GGGCTGCAGGAATTAACCATGC-3ʹ

Specific sequences for *Atg18* and *Wdr45l*:

5ʹ-Flanking region primer forward:

*Atg18*: +GTATTGCAGTTGGTACACCAGAAGG

*Wdr45l*: +CCTCACAATAGTAGCAGTAGTAATAAC

5ʹ-Flanking region primer reversed:

*Atg18*: +TGAAATTTGACTCTTATGCGCTTG

*Wdr45l*: +CTGTACCTGGACAAATTGCAC

3ʹ-Flanking region primer forward:

*Atg18*: +CGTTTCGAGTGATACTGGTAC

*Wdr45l*: +CATTCCAGCACACGAAGGTGC

3ʹ-Flanking region primer reversed:

*Atg18*: +TGGGTACACATCATCATTCTCAAC

*Wdr45l*: +GAAAATAGTTAGTTTGTAGAGTATCC

Yeast strain FY250 was transformed with approximately 100 ng of each amplified PCR fragments (2 flanking regions and the blasticidin cassette), and the plasmid pRS313 (GeneBank: U03439) previously digested with Xho1 and Xba1 and purified (see Fig. S1 for a schematic explanation).

Yeast transformants were selected in histidine-deficient media and DNA was isolated and used as template to amplify the whole disruption cassette with the most external 5ʹ and 3ʹ primers. Approximately 5 μg of the purified PCR product was electroporated in *Dictyostelium* WT cells. After seven days of blasticidin selection the transformants were plated on SM plates in association with *Klebsiella aerogenes* for clonal isolation. Mutants were identified by PCR as described in Fig. S2.

### Gene expression vectors for Atg18 and wdr45l and autophagic markers

Atg18 and Wdr45l coding sequence were amplified by PCR and cloned into PDM320 [88] (obtained from Dicty-stock center, ID-538). In this construct, a FLAG epitope is fused to the N terminus of the protein.

GFP-Atg18 and RFP-Atg18 were kindly provided by Dr. Jason King (Department of Biomedical Sciences, University of Sheffield, Sheffield, United Kingdom) [89].

Proteolytic cleavage assay was performed in cells expressing the construct GFP-PgkA. Cells were incubated with NH_4_Cl at the indicated concentrations and the levels of free-GFP were analyzed by western blot as previously described [66,67]. Vmp1-GFP was described previously [68]. The marker GFP-Atg8-RFP-Atg8ΔG was based on the mammalian marker previously described [65]. A synthetic DNA fragment (Integrated DNA Technologies) containing the sequence coding for Atg8a-RFP-Atg8a-ΔG was obtained. The codons of Atg8aΔG were extensively changed (keeping the same coding amino acids) to prevent recombination with the Atg8a-RFP sequence during subsequent cloning. This fragment was cloned in the BglII and SpeI sites of pDM317 (obtained from Dicty-stock center, ID-535), so the GFP is located upstream and in-frame with the first Atg8a.

### ER stress techniques

*Dictyostelium* cells were taken from the growing zone of plaques formed on Klebsiella lawns and resuspended in HL5. Cells were adjusted to 2 × 10^6^ cells/ml and 1 ml deposited in 6 MW as described previously [72]. Briefly, two samples were set up for each strain and one of them treated with tunicamycin at 2 μg/ml (Sigma-Aldrich, T7765) and the other with the vehicle dimethylsulfoxide (DMSO; Merck, 102,952). The plates were incubated in shaking condition for 17 h before protein isolation as described previously [72]. The levels of the protein CdcD were analyzed by western blot.

### Immunofluorescence and microscopy

For confocal microscopy *Dictyostelium* cells were transferred to Ibidi μ-Slide-eight-well chambers (Ibidi, 80,826) and directly observed *in vivo* (for cells expressing fluorescently-tagged proteins) or fixed for immunofluorescence detection. Fixation was performed for 15 min with 2% paraformaldehyde (Merck, 104,005) dissolved in 2x PBS (266 mM NaCl, 16 mM Na_2_HPO_4_, 4 mM KH_2_PO_4_, pH 7.4). After three washes with 1x PBS (133 mM NaCl, 8 mM Na_2_HPO_4_, 2 mM KH_2_PO_4_, pH 7.4) cells were blocked (blocking solution: 2% BSA [Sigma-Aldrich, A3059-10 G]; 0.5% NP-40 [Sigma-Aldrich, 74,385]; 1x PBS) for 30 min. Primary antibody incubation was left overnight at 4°C in blocking solution. The Atg8 antibody was kindly provided by Jason King (Department of Biomedical Sciences, University of Sheffield, Sheffield, United Kingdom) and used at 1:2000; the FLAG antibody (Cell Signaling Technology, 8146) was used at 1:1000. After three washes with 1x PBS cells were incubated 1 h at room temperature with the indicated secondary antibody conjugated to Alexa Fluorophores (Molecular Probes, A11034, A11035, A11029, A11030) in blocking solution at 1:500. Confocal images were acquired with an inverted Zeiss spectral LSM710 microscope.

### Western blot analyses

*Dictyostelium* cells (2x10^6^ cells) were centrifuged and washed once with PDF buffer. The cellular pellet was then resuspended with lysis buffer (10 mM Tris/HCl, pH 7.5, 150 mM NaCl, 0.5 mM EDTA, 0.5% NP-40, 0.05% SDS) supplemented with protease inhibitors (Sigma-Aldrich, P8340). Cells were kept in ice for 1 h and vortexed every 10 min. The sample was then centrifuged at 15,300 x g, and total protein concentration in the supernatant was determined by BCA (Thermo Fisher Scientific, 23,225). Protein extracts were subjected to SDS-PAGE separation and transferred to PVDF membranes (Millipore, IPVH00010). Protein detection was performed with the specified antibodies: anti-GFP (Sigma-Aldrich, G1544); anti-cdcD (VCP/p97) (rabbit anti-*Dictyostelium* CdcD kindly provided by Dr. Ludwig Eichinger (Center for Biochemistry, Institute of Biochemistry I, Medical Faculty, University of Cologne, 50,931 Cologne, Germany) [90]. MCCC1, loading control, was detected using streptavidin conjugated to horseradish peroxidase (HRP) (GE Healthcare Life Sciences, RPN1231-2 ml) [91]. Densitometric analysis of western blot images was performed with ImageJ software was used for quantitative analyses of western blot images.

### Statistical analysis

Mean values, Standard Deviation (SD) and significance were graphed and analyzed from at least three independent experiments. The significance of differences between groups was determined either by student t- test, or one-way, two-way ANOVA, depending on the number of variables of the experiment and additional tests were used as specified in the figure legends. When required, post hoc tests were applied as specified in figure legends.

## Supplementary Material

Supplemental Material

## Abbreviations

Atg: autophagy-related. BPAN: beta propeller protein-associated neurodegeneration; ER: endoplasmic reticulum; FYVE: Fab1, YOTB, Vac1 and EEA1; GFP: green fluorescent protein; KO: knockout; MCS: membrane contact site; PROPPIN: beta-propellers that bind polyphosphoinositides; PtdIns3P: phosphatidylinositol-3-phosphate; RFP: red fluorescent protein; TN: tunicamycin; VMP1: vacuole membrane protein 1; WDR45: WD repeat domain 45; WIPI: WD repeat protein domain, phosphoinositide interacting.
