# Supplementary material for: A Dictyostelium model for BPAN disease reveals a functional relationship between the WDR45/WIPI4 homolog Wdr45l and Vmp1 in the regulation of autophagy-associated PtdIns3P and ER stress
Source: Autophagy. 2021 Jul 27;18(3):661–77. doi: 10.1080/15548627.2021.1953262 (PMC9037511; doi:10.1080/15548627.2021.1953262)
Supplement: Supplemental Material [file KAUP_A_1953262_SM3498.zip › Supplementary_material_R4.docx]

**Supplementary material**

**
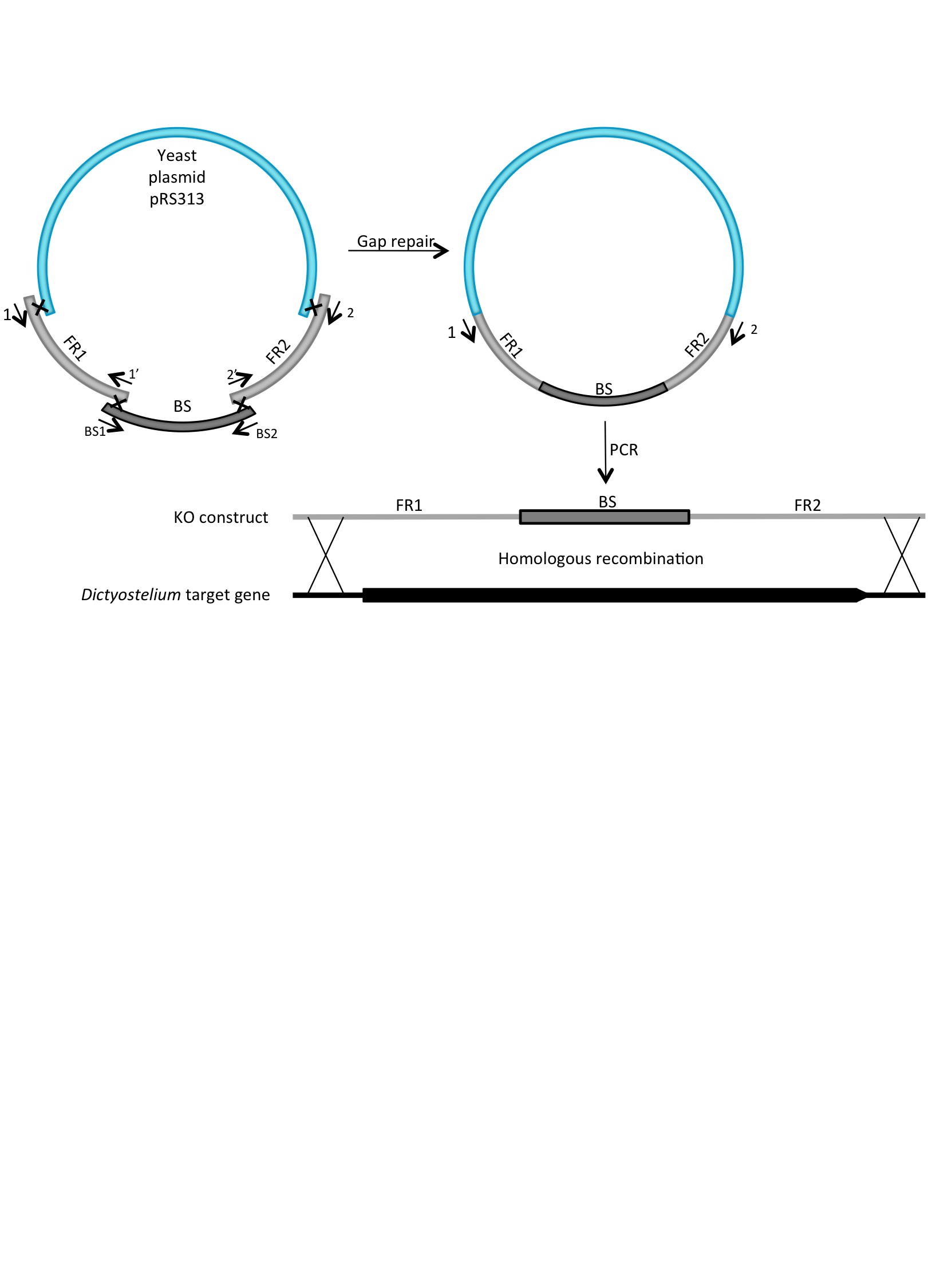
**

**Figure S1.** Generation of disruption constructs by gap repair. Schematic representation for the generation of disruption constructs by gap-repair. See the detailed protocol in materials and methods section. Yeast is transformed with the plasmid pRS313 and a mixture of PCR-amplified fragments corresponding to the BS-cassette (BS) and two flanking regions (FR1 and FR2) of the target gene. DNA from the yeast transformant strain is used as template for the amplification of the whole construct that will be then transformed in *Dictyostelium* cells.

**
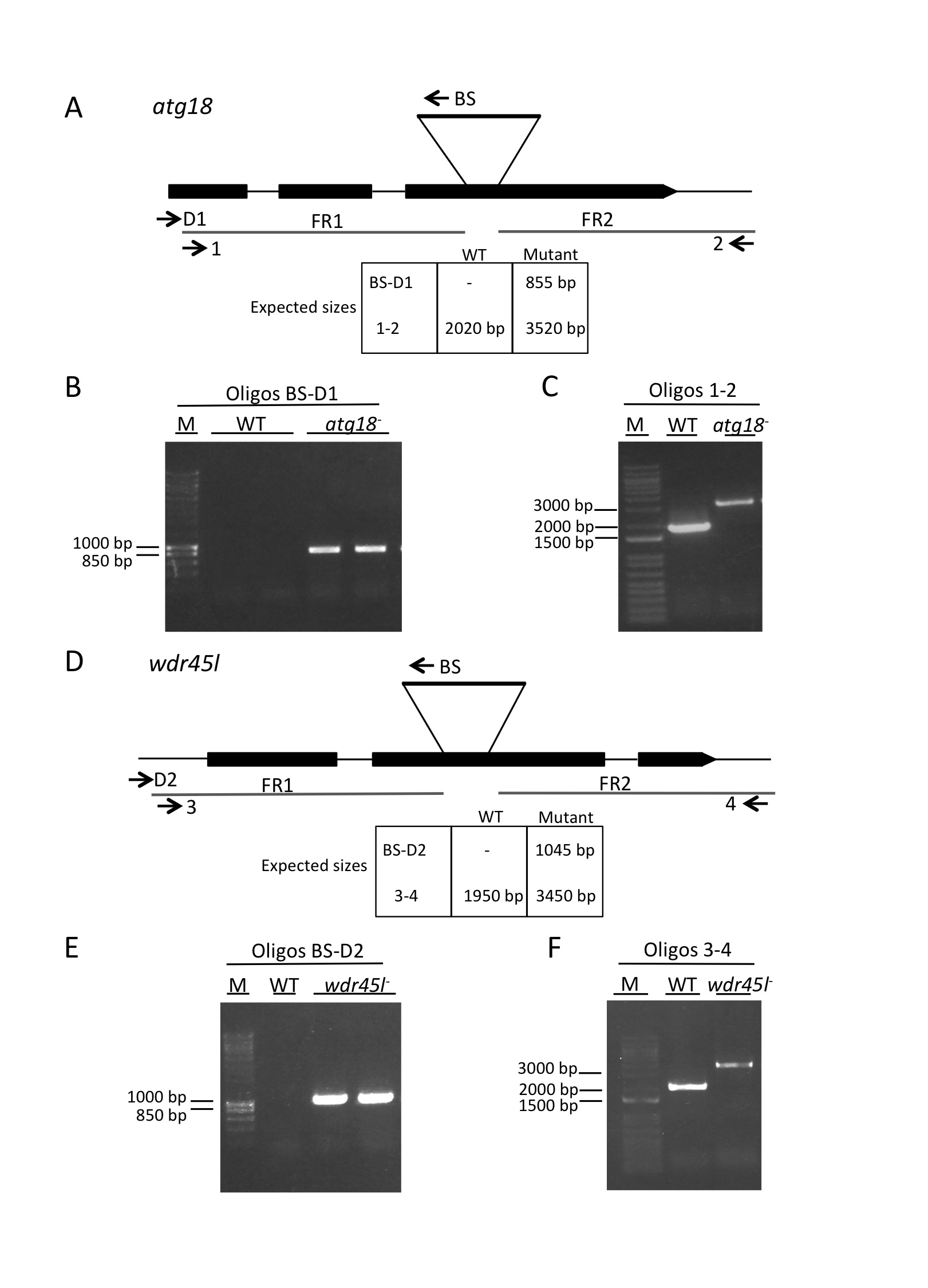
**

**Figure S2.** Disruption of atg18 and wdr45 in *Dictyostelium*. Schematic representation of *atg18* and *wdr45* loci (**A, D**), showing their coding sequence (solid black boxes), and non-coding regions (black lines). Grey lines mark the two flanking regions (FR1, FR2) used in the KO vector for homologous recombination. The BS cassette was located between the flanking regions and its final position depicted over the locus of the genes. A short deletion will be generated after homologous recombination between the flanking regions. Genotyping was performed by PCR (**B, E**), and the location of the oligonucleotides is indicated by arrows. The expected results of the PCR fragments for WT and mutated strains are indicated in the boxes below and an example of the results underneath.

**
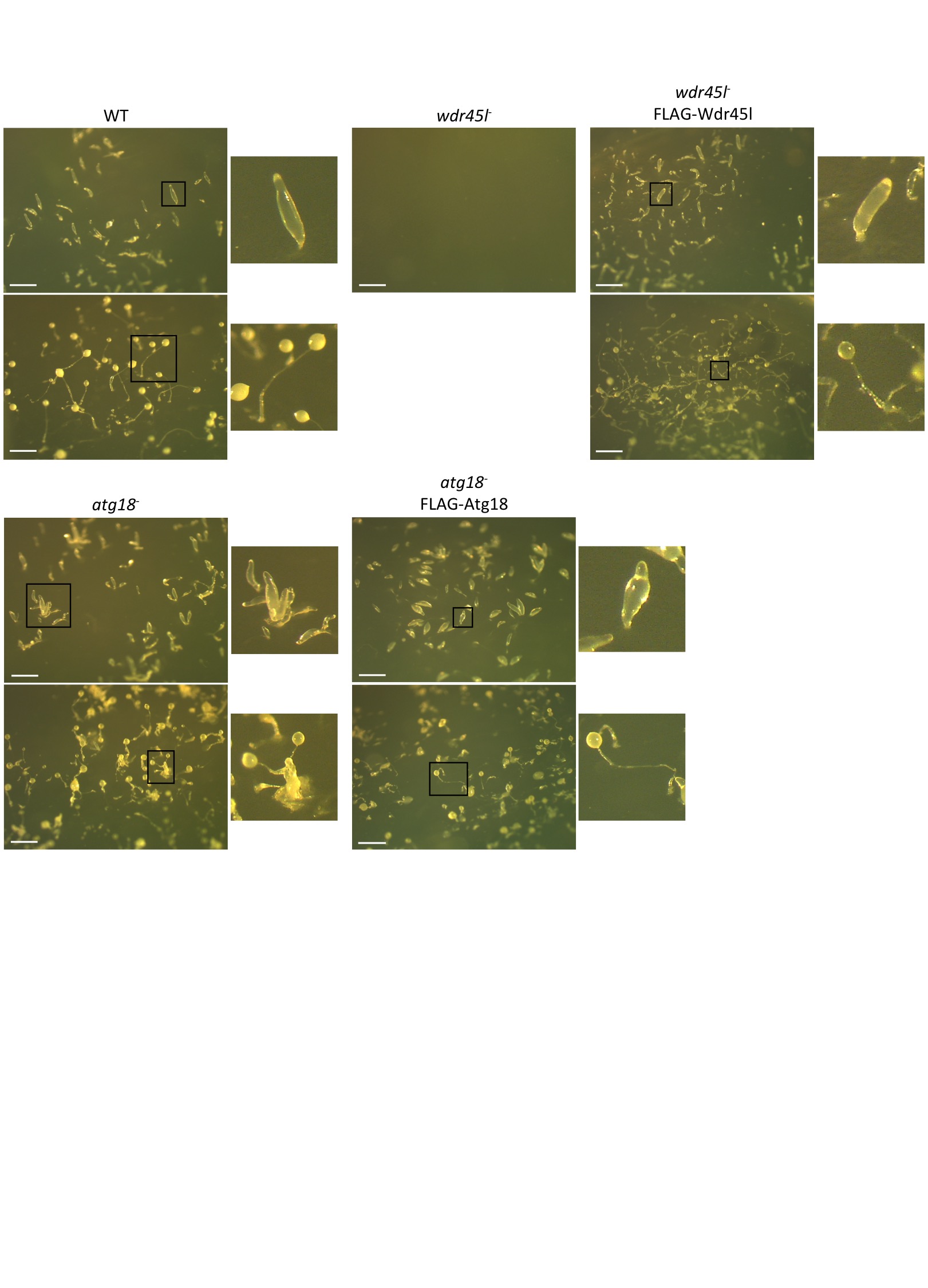
**

**Figure S3.** Developmental phenotype of *Dictyostelium* Atg18 and Wdr45l mutants in KK2 plates. Cells of the indicated strains were deposited in KK2 plates for synchronous development. Pictures were taken at the finger (14-16 h) and culmination (24-30 h) stages. Bar: 1 mm.

**
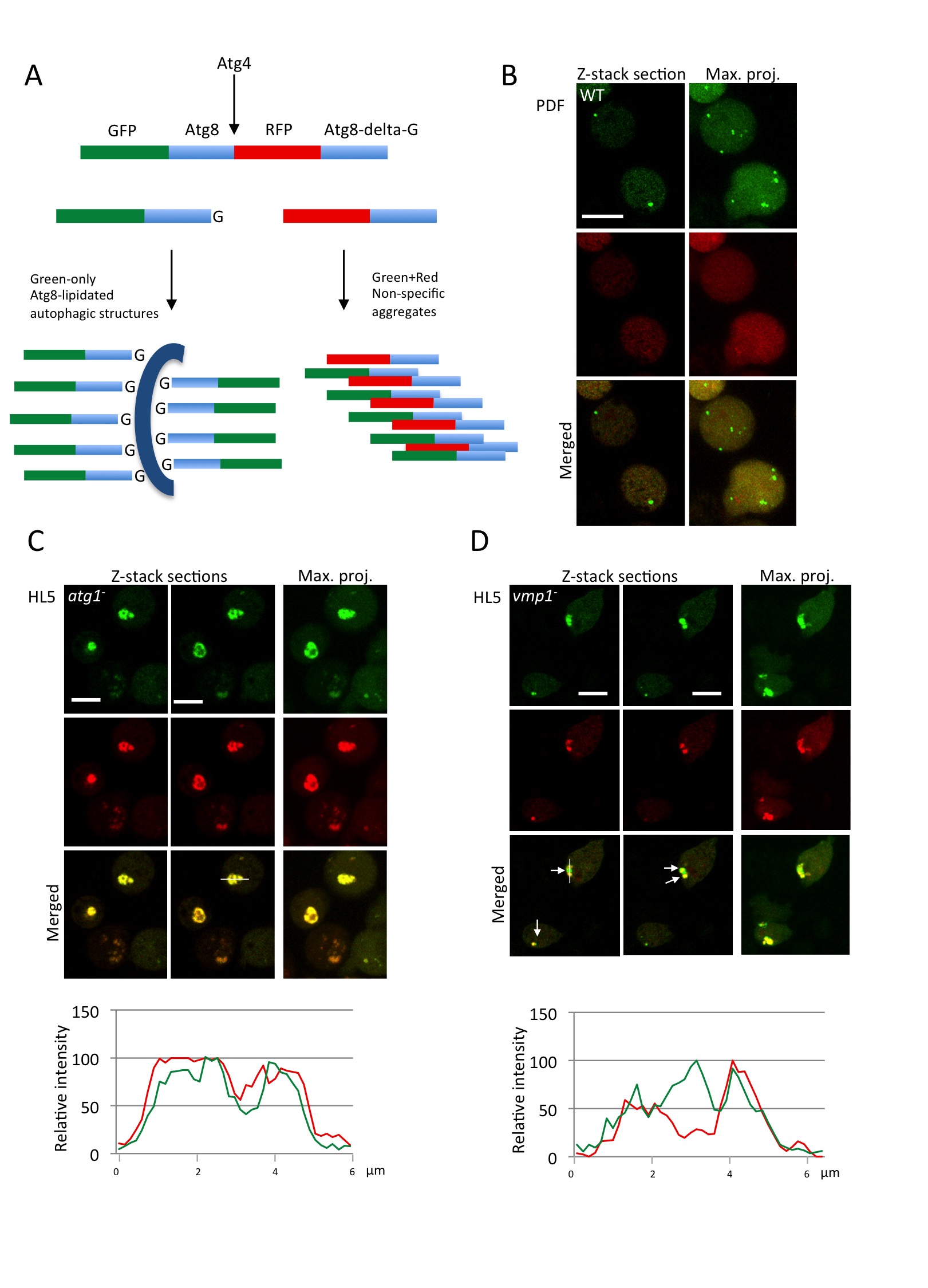
**

**Figure S4.** Validation of the autophagic marker GFP-Atg8-RFP-Atg8-ΔG in *Dictyostelium*. (**A**) The scheme illustrates the structure of the marker and the expected results under normal formation of autophagosomes in WT and the formation of protein aggregates that typically appear in *Dictyostelium* upon autophagic dysfunction. WT (**B**), Atg1 (**C**) and Vmp1 (**D**) mutant strains were transformed with the marker and analyzed by confocal microscopy *in vivo*. These mutant strains have a blockade in autophagy and were characterized previously (see main text). In WT a typical punctate pattern appears under starvation conditions (2 h in PDF). These structures do not contain red-fluorescence and are thus *bonafide* autophagic structures. Strains lacking Atg1 or Vmp1 show large accumulations of protein aggregates containing green+red fluorescence even under growth conditions (HL5), indicative of autophagic dysfunction. The Vmp1 mutant strain shows additional green-only structures associated with the aggregate (white arrows). Underneath, plot profiles of the aggregates were delineated following the white line depicted in the merged pictures. These analyses show the precise overlapping of green and red fluorescence for Atg1 mutant (**C**) and abnormal green-only structures in Vmp1 mutant (**D**). Bar: 10 μm.


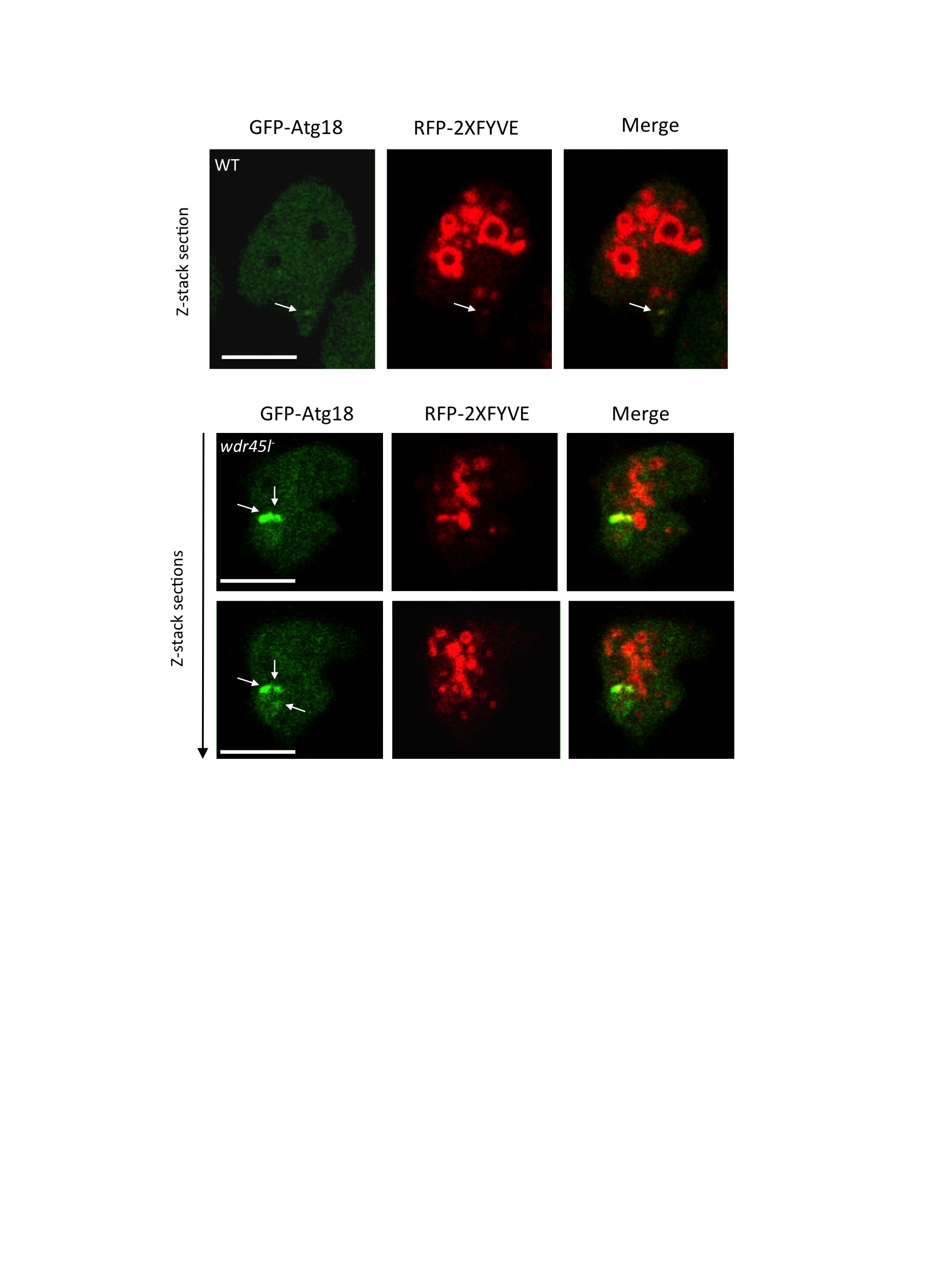


**Figure S5.** GFP-Atg18 colocalizes with the PtdIns3P reporter RFP-2xFYVE. WT and Wdr45l mutant cells were transfected with GFP-Atg18 and RFP-2XFYVE for colocalization studies. Stack sections are shown and colocalizations marked by arrows. Bar: 10 μm.

**
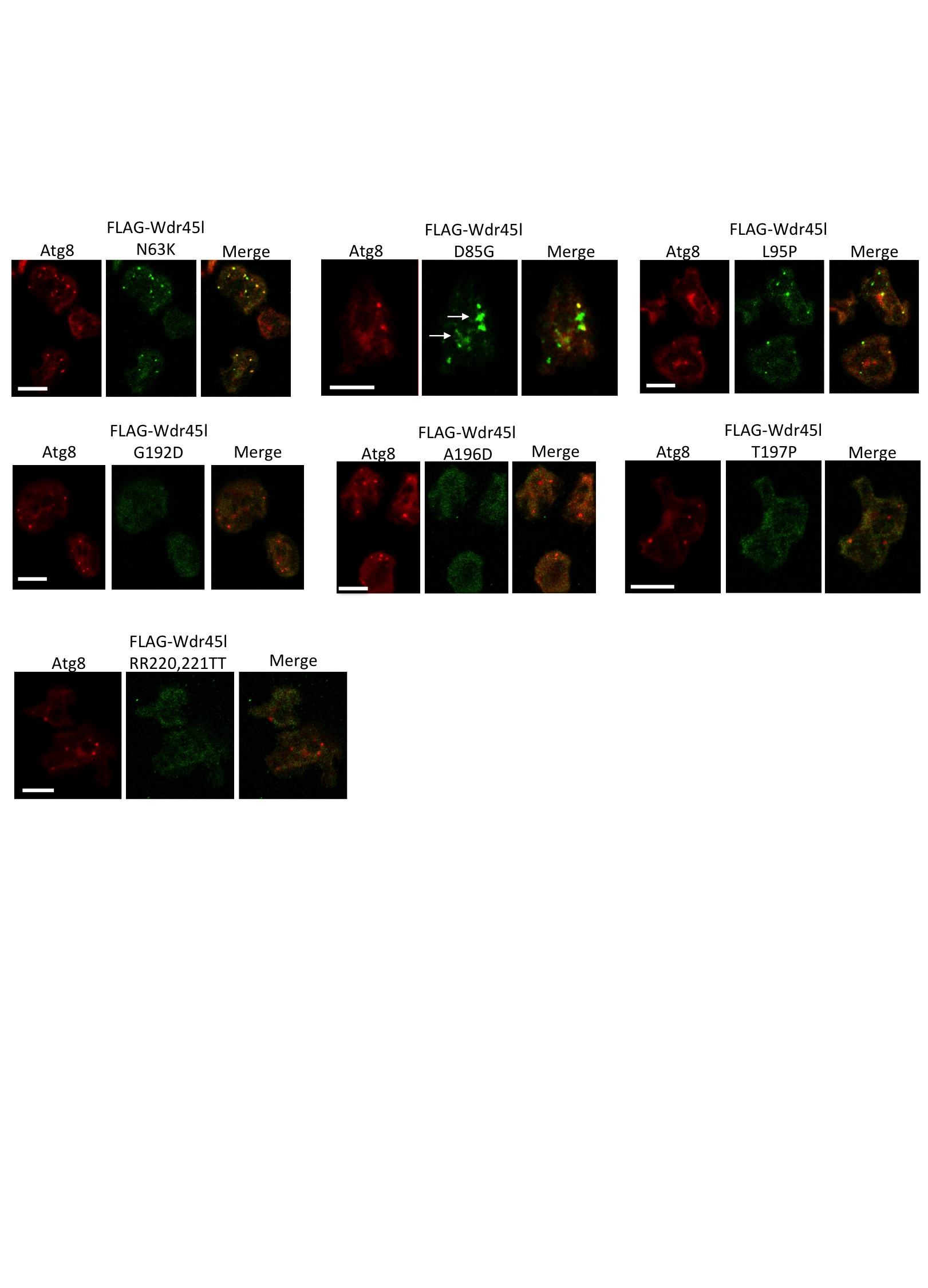
**

**Figure S6.** Subcellular localization of Wdr45l carrying pathological mutations. Mutant forms of Wdr45l fused to the FLAG epitope were expressed in WT, starved for 2 h and fixed for immunofluorescence with FLAG and Atg8 antibodies. Colocalization between both proteins was analyzed by confocal microscopy. White arrows in D85G mark aberrant accumulations of the protein. Three independent experiments were performed with similar results. In addition, DNA from independent clones of each transformation was isolated to verify "*a posteriori*" the presence of the expected mutations by sequencing PCR-derived *wdr45l* fragments. Bar: 10 μm.

**
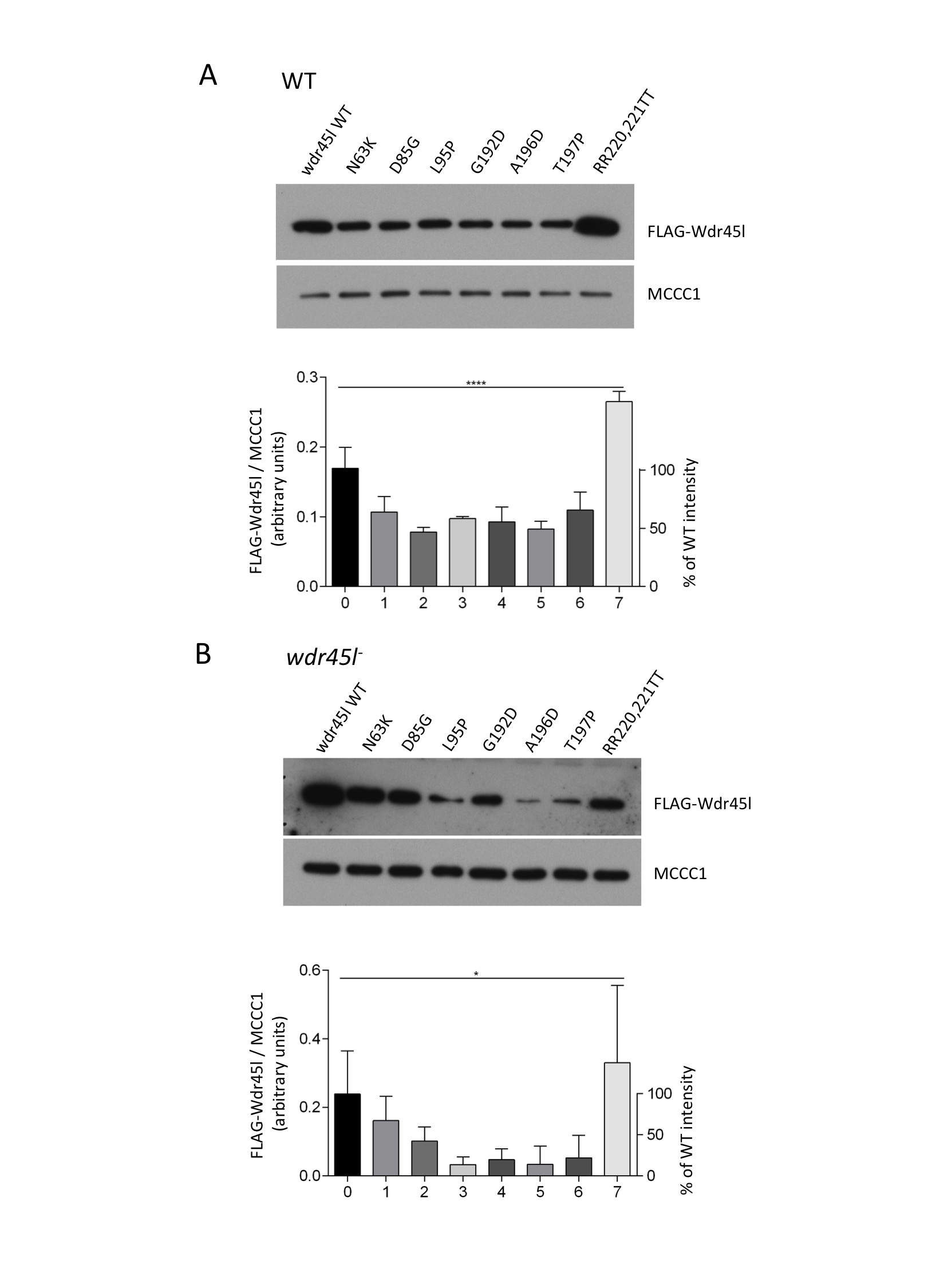
**

**Figure S7.** Western blot analysis of the expression of the FLAG-Wdr45l constructs. (**A**) Protein extracts from WT cells expressing WT and mutated Wdr45l were prepared and analyzed by western blot to detect the FLAG-epitope. Protein was isolated from cells growing in axenic media under selection with G418. Quantification of three experiments was analyzed by One-way ANOVA. (**B**) Protein extracts from Wdr45l mutant strain expressing the WT and mutated Wdr45l were isolated and analyzed by western blot to detect the FLAG-epitope. Protein extracts were prepared from cells growing on SM plates. Quantification of three experiments was analyzed by One-way ANOVA. Significance of differences are denoted by asterisks: * p<0.05; *** p<0.001.


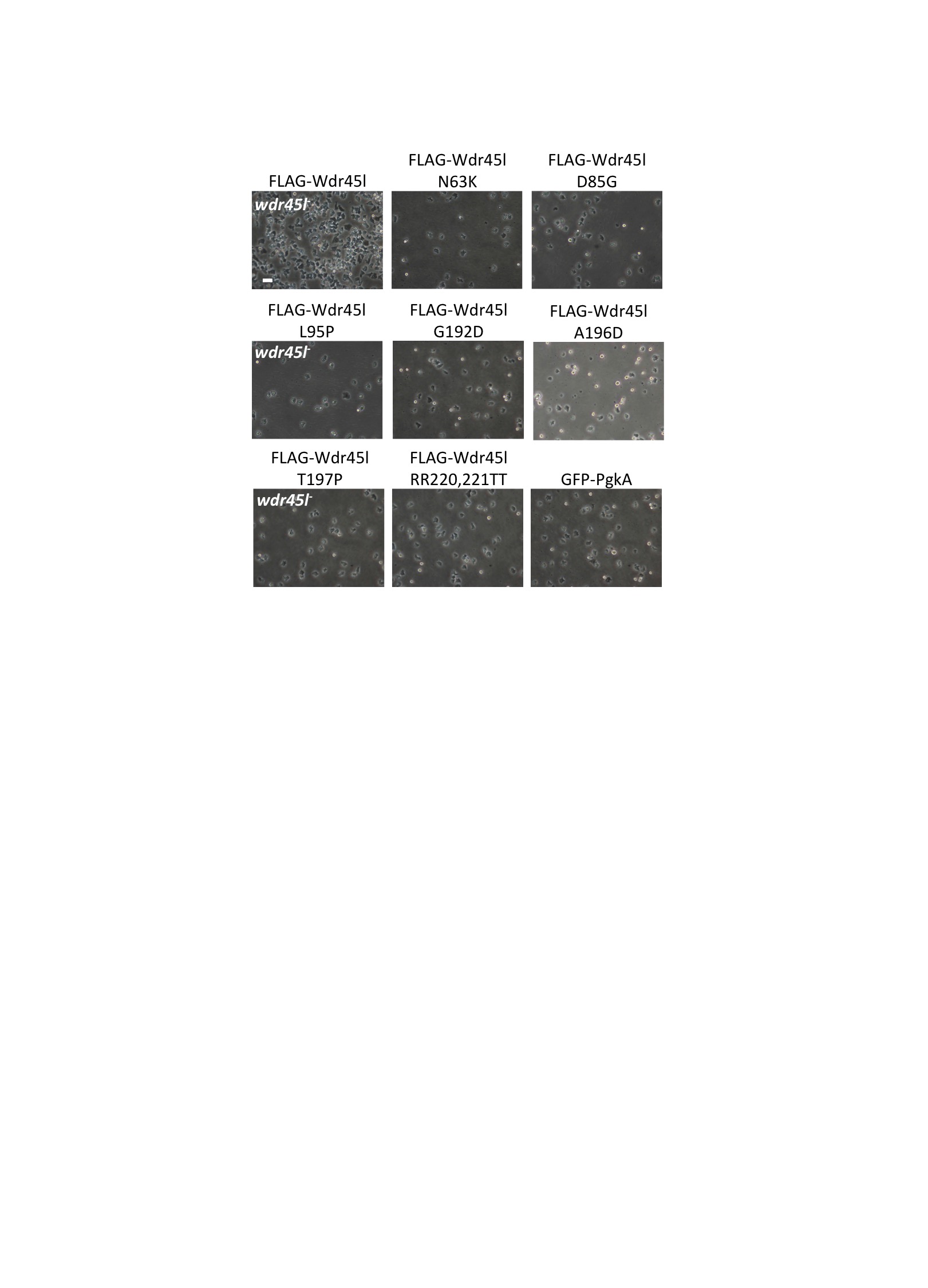


**Figure S8.** Functional analysis of the mutated forms of Wdr45l. WT and mutant forms of Wdr45l fused to the FLAG epitope were transformed in the Wdr45l mutant. After 7 d under selection, cells were observed by phase microscopy to assess growth and cell morphology. Two independent transformations were performed with similar results. In addition, DNA was isolated from independent clones of each transformation to verify "*a posteriori*" the presence of the expected mutations by sequencing PCR-derived *wdr45l* fragments. Bar: 10 μm.

**Legends for supplementary movies**

**Movie 1 and movie 2.** WT (movie 1) and Wdr45l mutant (movie 2) grown in HL5 (from the experiments shown in **Fig. 5A**) were visualized in an inverted microscope (Nikon Eclipse TS199) and recoded with a Leica camera MC170HD. Video displayed 3 times faster than real time.

**Movie 3 and movie 4.** Video-lapse of WT (movie 3) and Wdr45l mutant (movie 4) expressing GFP-Atg18. Video displayed 15 times faster than real time.

**Movie 5.** Video lapse of Wdr45l mutant expressing Vmp1-GFP. Video displayed 15 times faster than real time.
